# Psychologists in primary care: A scoping review exploring the views and experiences of patients and professionals on psychology provision in primary care

**DOI:** 10.1111/aphw.70178

**Published:** 2026-07-09

**Authors:** Kirstie McClatchey, Aidan Jordaan, Patrick Robinson, Emma Kinley, Hannah Dale, Liz Steed, Hilary Pinnock

**Affiliations:** ^1^ Directorate of Public Health NHS Tayside, Kings Cross Hospital Dundee UK; ^2^ School of Medicine University of Dundee Dundee UK; ^3^ Edinburgh Medical School The University of Edinburgh Edinburgh UK; ^4^ School of Psychology, Faculty of Health Liverpool John Moores University Liverpool UK; ^5^ Wolfson Institute of Population Health Queen Mary University of London London UK; ^6^ Usher Institute The University of Edinburgh Edinburgh UK

**Keywords:** behavioural health, general practice, primary care, psychologist, psychology

## Abstract

Psychologists in primary care have the potential to deliver interventions targeting behavioural risk factors for long‐term conditions and can support self‐management of physical and mental health conditions. The British Psychological Society recommends that psychology be embedded routinely into primary care. However, this is not yet implemented. We aimed to explore how the role of psychology professionals has been co‐located/integrated within primary care and synthesise views and experiences of patients, primary care staff and psychology professionals to inform future practice. A scoping review was performed using CINAHL, MEDLINE, PsycINFO and Google Scholar. The searches included articles published in English from 2010 onwards. Reference lists of included articles were screened, and forward citation tracking was carried out to identify further articles. Seventy‐two studies were included, and patients and primary care staff described psychology professional provision in primary care as positive. Having co‐located/integrated psychology professionals has the potential to increase and/or improve access to care, improve patient knowledge/skills and reduce primary care staff workload and stress. There were development opportunities for primary care and psychology staff, and quality of care was described as improved. Psychology professionals, although satisfied, reported challenges (e.g. role demands and organisational challenges) and training needs. The review discusses practical suggestions, implementation considerations and further research needs.

## INTRODUCTION

A wide range of public health issues have the potential to be supported in primary care. Long‐term conditions (LTCs) (e.g. cardiovascular diseases, chronic respiratory diseases and diabetes) are estimated to account for 89% of all deaths in the United Kingdom (World Health Organization [WHO], 2018). Additionally, it is estimated that one in six UK adults meet the criteria for a common mental health disorder (McManus et al., 2016). Recent UK statistics show that the number of people economically inactive because of long‐term sickness has risen to over 2.5 million people (Office for National Statistics, 2023). Further, costs to the healthcare system are substantial—12%–18% of all National Health Service (NHS) expenditure on LTCs is linked to poor mental health (between £8 billion and £13 billion in England each year) (Naylor et al., 2012).

The largest volume of NHS activity is in primary care (The King's Fund, 2023). A responsibility of primary care providers is to engage patients in health promotion such as modifying harmful health behaviours (e.g. smoking, unhealthy weight and harmful alcohol use), with general practitioners (GPs) being required to offer ‘relevant health promotion advice’ to patients under the General Medical Services contract (Beaney & Allen, 2019). GPs and nurses are unlikely to have specialist training in behaviour change compared with, for example, health psychologists (the only healthcare professional [HCP] group trained to a doctoral level in behaviour change interventions [The British Psychological Society, BPS, 2020]).

GPs are faced with unmanageable workloads, with GPs in England seeing on average 37 patients per day (Pulse, 2023), far higher than the recommended safe level of 25 for routine contacts, or 15 for long‐term, complex or mental health conditions (British Medical Association, 2018). There is, at present, a high level of GP burnout (Biddle et al., 2023; Karuna et al., 2022). The GP workforce is facing a retention crisis, and there are suggestions to introduce alternative models of care (e.g. multidisciplinary team‐based models and the Nuka System of Care) (BMA Scotland, 2023; Owen et al., 2019).

Co‐located or integrated (Brown et al., 2021) psychologists in primary care can potentially deliver health promotion‐focused interventions, support those at risk of experiencing mental health problems (e.g. people living with LTCs) who, without support, may eventually require referral to secondary care services, support people to self‐manage their conditions, which has the potential to reduce healthcare use and improve quality of life (Hodkinson et al., 2020; Pinnock et al., 2017), and support prevention efforts and treatment of LTCs for those who are at greater risk of poor health (e.g. people living with severe mental illness) (O'Connor et al., 2023).

Evidence suggests that the provision of co‐located/integrated psychology in primary care can be of benefit to patients by increasing access to care (Miller‐Matero et al., 2015; Miller‐Matero et al., 2016); improving patient outcomes (Chomienne et al., 2011; Miller‐Matero et al., 2021); supporting families of patients (Barman & Paulson, 2022); and reducing pharmaceutical expenditure (Falanga & Pillot, 2020; Unwin et al., 2023). Further, co‐location/integration of a psychology professional in primary care could also help reduce GP workload (Chomienne et al., 2011; Farmanova et al., 2017) and personal stress levels of clinicians (Miller‐Matero et al., 2016). There is limited review evidence focused on care delivered by a psychology professional explicitly co‐located or integrated in the primary care team, and it has been suggested that it may be helpful to explore staff experiences and views to understand challenges when delivering psychological interventions in primary care (Finazzi & Macbeth, 2022).

### Aims of the scoping review

There have been international efforts to integrate psychology provision into primary care (e.g. in Norway, where primary health services are now required to provide psychologists [Kaspersen et al., 2019]). Recently, the BPS has recommended that psychology should be embedded in UK primary care, with at least one psychologist per primary care practice population of 50,000 patients (BPS, 2022a). However, this is yet to be routinely implemented. Given the potential for widespread implementation of psychologists in UK primary care practices, it is important that stakeholders' views and experiences of this provision are understood and areas where further research is needed are identified in order to develop an effective implementation strategy. We therefore aimed to scope the literature exploring how psychology professionals have been co‐located/integrated in primary care to synthesise the views and experiences of patients, primary care staff and psychology professional groups on this role.

## MATERIALS AND METHODS

### Study design

A scoping review was chosen as we considered it likely that psychologists will have been located within primary care not only as part of a formal research process but also as part of quality improvement initiatives or clinical pilot studies, and we felt it important to learn from these experiences and research publications. We aimed to identify areas where further research would be useful prior to developing an implementation pathway (Munn et al., 2018). The review follows scoping review guidance (Peters et al., 2020), and the reporting follows the Preferred Reporting Items for Systematic Reviews and Meta‐Analyses extension for Scoping Reviews (PRISMA‐ScR) checklist (Tricco et al., 2018).

### Eligibility criteria

The PICo (Population, Phenomena of Interest, and Context) framework (Lockwood et al., 2015) was utilised to frame the inclusion and exclusion criteria (Table 1). Psychology professional groups were included broadly in the review; this included Health and Care Professions Council (HCPC)–registered psychologist roles (BPS, 2022b; HCPC, 2025), the wider psychological workforce (BPS, 2022b) and their international equivalents (e.g. behavioural health consultants). Although the psychologist role in primary care is most likely suited to a health psychologist (a protected title and modality of practitioner psychologists as registered by the HCPC in the UK) due to their expertise in behavioural support and ability to support common mental health problems within the context of primary care (e.g. where an individual has not been referred to secondary care services or is on a waiting list), we recognise that other psychology professional disciplines (e.g. clinical and counselling) can deliver behavioural support through a range of training routes. Psychology professional groups that were both co‐located and integrated were included, as although these are conceptually distinct (Brown et al., 2021), embedding psychology into primary care relies on co‐location as a mechanism to promote and facilitate integrated care.

**TABLE 1:** Scoping review inclusion and exclusion criteria.

	Inclusion	Exclusion
Population	Psychology professional groups (e.g. psychologist, behavioural health consultant, mental health worker and professional qualified in mental/behavioural health) co‐located/integrated in primary care, delivering psychological support for a health condition.	Psychology professional groups not co‐located/integrated in primary care; other professional groups delivering psychological support in primary care (e.g. GPs, nurses and psychiatrists delivering psychological support); non‐professional groups (e.g. lay counsellors and peer‐led support).
Phenomena of interest	The views and experiences of co‐located/integrated psychological support in primary care from patients, primary care staff (GPs, nurses, administrative staff, etc.) and professional groups delivering the psychological support (e.g. psychologists).	Studies not providing information on the views and experiences of patients, primary care staff and professional groups delivering psychological support in primary care (e.g. psychologists).
Context	Primary care settings (that are relevant to and can be applied to a UK primary care context), where psychological support is provided directly in a primary care setting (this includes remote consultations, e.g. telephone and video, when the professional is co‐located/integrated in primary care).	Settings that are not relevant to/cannot be applied to UK primary care settings (e.g. paediatric or veteran primary care practices); secondary or tertiary care settings; or any other settings that are not primary care (e.g. where a patient has been referred from primary care to a service outside of primary care).
Study design	Any study design (e.g. qualitative, quantitative, mixed‐method, reports and conference abstracts) that includes the views and experiences of patients, primary care staff and professional groups delivering psychological support in primary care.	Study designs that do not include any views of patients, primary care staff and professional groups delivering psychological support in primary care (e.g. psychologists). Book chapters, letters and commentaries will be excluded.

Abbreviation: GPs, general practitioners.

To capture recent literature likely to be applicable to contemporary practice, studies published from 2010 onwards were included. Studies not published in English were excluded.

### Information sources and search strategy

Relevant health and social science databases (CINAHL [EBSCO], MEDLINE [Ovid], PsycINFO [Ovid] and Google Scholar) were searched in June 2023. Google Scholar was utilised given its strength in finding grey literature, its effectiveness in providing comprehensive citations and its ability to complement citations from other databases (Martín‐Martín et al., 2018; Martín‐Martín et al., 2021). Example search terms included ‘psychology’, ‘primary care’ and ‘general practice’. Search terms pertaining to views and experiences and study design were not included but were instead considered during the inclusion/exclusion process. See File S1 for an example search term for the database PsycINFO. Additional searches of the reference lists of included studies were conducted. Prior to submission for publication, forward citation tracking was carried out on all included studies (26 July 2025), which is recognised as an efficient approach to updating reviews (Cantrell et al., 2022).

### Study selection

The final search results were exported into Covidence, which provided an initial deduplication, with further deduplication conducted using the SRA Deduplicator tool (Clark et al., 2020). Titles and abstracts were screened by two reviewers (A. J. and P. R.) in Covidence, overseen by a third reviewer (E. K.). A fourth reviewer resolved any conflicts (K. M.). Full texts were screened using the same process.

### Data charting

Relevant data were charted in a table to record the findings of the included studies (File S2). The charting table was developed by three reviewers (A. J., K. M. and P. R.). Two reviewers (A. J. and P. R.) independently charted the data from the included studies, discussed the results with K. M. and updated the data‐charting table where necessary (e.g. to cover the diversity of study types) in an iterative process. Key elements captured in the charting table include the psychology professional group (e.g. psychologist, behavioural health consultant etc.), the service/intervention delivered and key findings. We did not extract outcome data but only information that pertained to the views and experiences of patients and/or professionals on psychology provision in primary care.

### Synthesis of results

Data were summarised in a descriptive format (narratively described), and a table and figure were produced to illustrate the results.

## RESULTS

**FIGURE 1:**
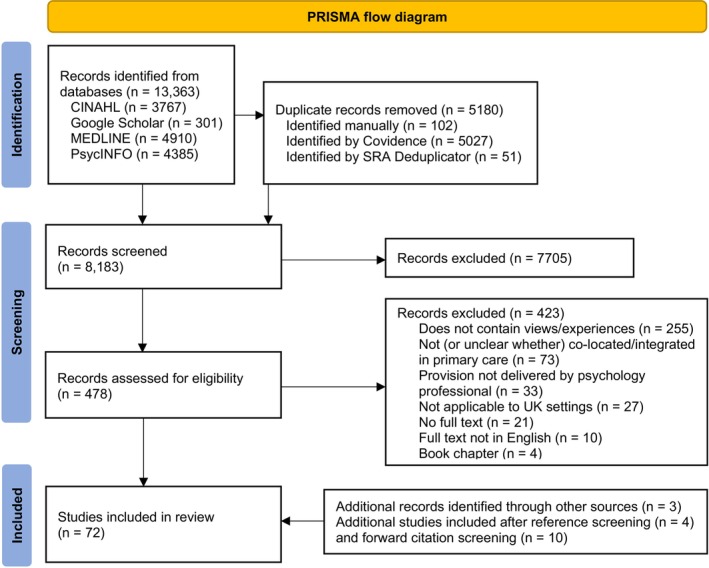
PRISMA flowchart for the study selection process.

The database search (2023) generated 8183 sources after duplicates were removed. After screening and reviewing against the inclusion/exclusion criteria, 72 sources were included; this includes an additional 17 identified through other sources (e.g. conference abstracts by co‐authors) (*n* = 3), reference list screening (*n* = 4) and updating prior to publication (2025) using forward citation (*n* = 10). Figure 1 displays a PRISMA flowchart.

### Study characteristics

Included study characteristics can be found in File S2. Studies were published between 2010 and 2025 and were conducted in the United States (*n* = 34), the United Kingdom (*n* = 13), Canada (*n* = 4), Australia (*n* = 3), New Zealand (*n* = 3), Spain (*n* = 3), Ireland (*n* = 2), Italy (*n* = 2), Netherlands (*n* = 2), Sweden (*n* = 2), Hungary (*n* = 1) and Norway (*n* = 1). The location of the two studies was not reported. A mixture of qualitative (*n* = 31), quantitative (*n* = 8), mixed‐methods (*n* = 26) and other study designs (e.g. reviews) (*n* = 6) were included. One study did not report the study design but provided quantitative findings.

The job title of co‐located/integrated psychology professional groups included psychologist (*n* = 34) (including clinical, health and clinical health), behavioural health counsellor/consultant/provider/staff (*n* = 21), psychological well‐being practitioner (*n* = 2), advanced clinical psychology doctoral/graduate student (*n* = 2), pre‐doctoral clinical psychology graduate student (*n* = 1), graduate student intern in clinical psychology (*n* = 1), early career counselling psychologist (*n* = 1), trainee associate psychological practitioner (T/APP; *n* = 1), mental health professional (*n* = 1) and therapist (*n* = 1). Six studies described multiple psychology groups, and one study did not report the title.

Psychology services addressed mental health problems (*n* = 18), physical health problems (*n* = 19) or a combination (*n* = 19). Specific health conditions were not reported for 16 studies. The health conditions included (but were not limited to) anxiety, bipolar disorder, cancer, chronic obstructive pulmonary disease (COPD), chronic pain, depression, diabetes, fibromyalgia syndrome, heart disease, medically unexplained symptoms, obesity, schizophrenia, sleep, smoking, stress, substance use and suicidal behaviour. Broadly, interventions delivered by psychology professional groups in primary care included brief interventions, acceptance and commitment therapy, behavioural activation, cognitive behavioural therapy (CBT), mindfulness, motivational interviewing, psychoeducation and self‐help (see File S2). Of papers that reported the number of sessions a psychology professional delivered with an individual patient, this ranged from 1 to 40 (see File S2).

### Synthesis of results

**FIGURE 2:**
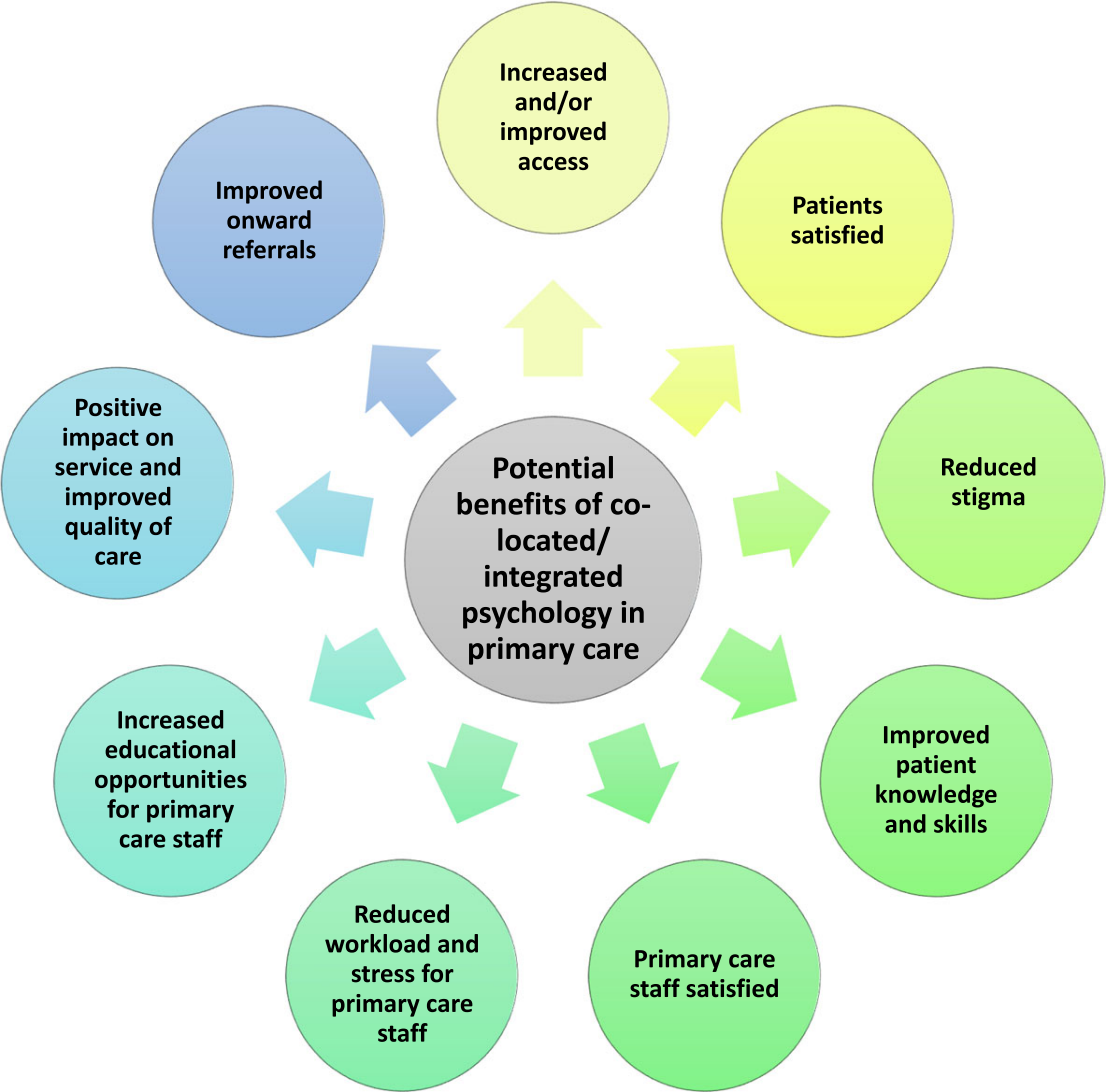
Potential benefits of a co‐located/integrated psychology professional in primary care.

Three main categories (the patient experience, professional experiences and organisations) with accompanying subcategories were extracted from the data, which are displayed in Table 2 and reported below. The synthesis found that views and experiences of patients, primary care staff and psychological professional groups appear to be positive, with many studies highlighting a potential benefit of having a psychology professional co‐located/integrated in a primary care practice. This provision does not come without its challenges, and these are discussed in the relevant categories. Further, a number of sources provided considerations for practice, which are detailed in the relevant considerations subcategories. Figure 2 visually displays potential benefits of having a co‐located/integrated psychology professional in primary care.

**TABLE 2:** Categories and subcategories of synthesised data.

Main category	Subcategory	Subcategory description/examples
The patient experience	Increased and/or improved access	Psychology provision in primary care has potential to improve access to care (e.g. patients able to be seen in familiar, local practice).
	Patient satisfaction	Patients appeared satisfied with the psychological and behavioural support provided.
	Reducing stigma	Access to support in a familiar location can reduce stigma.
	Patient knowledge and skills	Patients may acquire new knowledge and skills and utilise the skills they have learned.
	Relationship with psychology professionals and primary care providers	Patients described personal qualities about the psychology professionals and the impact on relationships with medical professionals.
	Challenges and issues raised by patients	Issues around, e.g. the care they received and the flexibility of the approach.
	Patient considerations	More support for patients in primary care and further training for the psychology professional groups.
Professional experiences	Primary care staff perspective	Primary care staff appeared satisfied with having a psychology professional in primary care, and psychology provision had a positive impact.
	Psychology professional perspective	Psychology professionals were satisfied and identified challenges and training needs.
	Working together	Primary care staff and psychology groups discussed barriers and facilitators to interprofessional collaboration.
	Professional considerations	Increased provision, psychology professional‐specific considerations and communication and collaboration considerations.
Organisations	Information sharing and communication	Communication can be facilitated by the electronic health record (EHR), open door policies, warm handoffs, etc.
	Referrals	Impact on referral practices.
	Organisational challenges	Access and appointments, and the physical environment and equipment.
	Organisational considerations	Information sharing and communication, referrals and appointment considerations.

### The patient experience

#### Increased and/or improved access

Sources described how a co‐located/integrated psychology professional in primary care increased and/or improved access to care (Austin, 2012; Bassilios et al., 2014; Beacham et al., 2012; Berkel et al., 2019; Berry, 2020; Budd et al., 2022; Chomienne et al., 2011; Dath et al., 2014; Farmanova et al., 2017; Hannah et al., 2012; Hartley et al., 2022; Hepworth et al., 2015; King et al., 2013; Knowles et al., 2015; Miller‐Matero et al., 2016; Perreault et al., 2023; Seierstad et al., 2017; Tierney & Merrick, 2022; Vickers et al., 2013; Yasmin‐Qureshi & Ledwith, 2020). Patients appreciated the local access (Raybould, 2019), and one study described how 100% of respondents to a patient satisfaction survey reported they would prefer to receive therapy at their GP clinic rather than go to a mental health service (Dath et al., 2014).

#### Patient satisfaction

Patients were generally satisfied with receiving psychological interventions, services and/or support in primary care (Allen et al., 2025; Angantyr et al., 2015; Arfuch et al., 2021; Brooks et al., 2016; Budd et al., 2022; Caballol Angelats et al., 2023; Cordella et al., 2016; Dath et al., 2014; Dueweke, 2019; Durcan, 2020; Enos, 2016; Falanga & Pillot, 2020; Gomez, 2017; Guarino, 2020; Hannah et al., 2012; Hard et al., 2015; Hartley et al., 2022; Hepworth et al., 2015; Holt et al., 2022; Hunter et al., 2018; Malins et al., 2016; McElvaney & Timulak, 2013; Meguro, 2018; Perreault et al., 2023; Ross et al., 2019; Tierney & Merrick, 2022; Turgesen, 2010; Vogel et al., 2012). However, one study found that patients with mild‐to‐moderate health conditions (physical and/or mental) may experience greater satisfaction with behavioural health integrated into primary care than patients with multiple and/or severe conditions (Koehler et al., 2020). Patients also appreciated the option of psychological rather than pharmacological support (Berry, 2020; Dueweke, 2019; Miller‐Matero et al., 2019).

#### Reducing stigma

Patients and primary care staff described the provision of psychology in primary care as less stigmatising (Austin, 2012; Berry, 2020; Dath et al., 2014; Enos, 2016; Holt et al., 2022; Knowles et al., 2013; Knowles et al., 2015; Monnickendam et al., 2025; Sallay et al., 2023; Seierstad et al., 2017), and patients spoke of valuing the opportunity to be heard without judgement (Malins et al., 2016).

#### Patient knowledge and skills

Patients reported acquiring new skills and practical strategies (Allen et al., 2025; Malins et al., 2016; Sallay et al., 2023), for example, improved coping skills and symptom self‐management (Arfuch et al., 2022; Raybould, 2019). Further, patients reported using skills they had learned (Brooks et al., 2016) and felt more able to manage their physical symptoms and mental health problems (Dath et al., 2014; Lorentzatou et al., 2021).

#### Relationship with psychology professionals and primary care providers

Broadly, patients were satisfied with the professionals (Arfuch et al., 2021). Patients described positive qualities about the psychology professionals (e.g. kind, caring, warm, empathic, non‐judgmental, knowledgeable and trusting) (Gomez, 2017; Yasmin‐Qureshi & Ledwith, 2020). However, one study reported that patients with mild‐to‐moderate conditions (physical and mental health) made faster and stronger relationship bonds with psychologists, and those with more serious conditions were more critical of their therapist relationships (Berry, 2020).

Patients felt that psychologists were better trained than medical practitioners to handle their psychological problems, but seeing the psychologist did not adversely affect their relationship with their doctor (Chomienne et al., 2011). When exploring joint physician and psychologist consultations, it was found that patients were four times more likely to perceive a personal relevance (e.g. the psychologist could help with discomfort in particular moments such as family, emotional and working life) than those who consulted the physician alone (Cordella et al., 2016). Further, patients reported better collaboration (adherence) with their GPs (Sallay et al., 2023), and patients were positive about the enhanced communication between the professionals, who previously had worked in isolation from each other (non‐collaboratively) (Knowles et al., 2015).

#### Challenges and issues raised by patients

Psychology provision in primary care is not without its challenges. Timing of provision can be important to patients, whereby the ability to engage with psychology can be affected by life circumstances (Raybould, 2019). Patients also had reservations about the practices' lack of confidentiality, as people in the local community knew each other (Caballol Angelats et al., 2023). Provision could also be constrained—one study reporting on an Improving Access to Psychological Therapies (IAPT) service model found that patients described their therapists as inflexible and rigid (likely mediated by the manualised CBT approach) (Yasmin‐Qureshi & Ledwith, 2020). A further study reported that the psychology programme offered in primary care was limited in its timeframe (Arfuch et al., 2021).

#### Patient considerations

Patients wanted more widely available psychology support in primary care (Brooks et al., 2016; Kierans & Byrne, 2010; Malins et al., 2016; Miller‐Matero et al., 2019). Interestingly, in one study, patients acknowledged limitations in support that the professionals (T/APPs, UK NHS Band 4) were able to provide and suggested that developing further skills may be beneficial (Budd et al., 2022).

### Professional experiences

#### Primary care staff perspective

##### Primary care staff satisfaction

Primary care staff appeared satisfied with having a psychology professional and the service they provided, and they felt they were important to the practice (Brooks et al., 2016; Fletcher, 2021; Hannah et al., 2012; Hunter et al., 2018; Meguro, 2018; Miller et al., 2023; Miller‐Matero et al., 2016; Raybould, 2019; Ross et al., 2019; Tierney & Merrick, 2022; Torrence et al., 2014; Turgesen, 2010; Vogel et al., 2012). However, a study discussed one of seven physician participants being dissatisfied with the integrated model of care, as it offered little in the way of consultation or timely assistance with complex patients (Austin, 2012).

##### Impact of psychology provision

Overall sources reported primary care staff as positive about the psychology professionals, suggesting that it was potentially important and beneficial to have a psychology provider on‐site (Johanson, 2021; Miller et al., 2023; Raybould, 2019). Staff reported that behavioural health clinicians effectively help patients address their mental and physical health problems (Johanson, 2021; Torrence et al., 2014), and primary care staff felt the psychology service was beneficial and had a positive impact on patients (Lorentzatou et al., 2021; Meguro, 2018). Doctors had good working relationships with psychologists (Chomienne et al., 2011), and primary care staff welcomed the integration of psychology professional groups (Chomienne et al., 2011; Holt et al., 2022; Miller et al., 2023).

Most sources found that psychology professionals in primary care had a positive impact on the service, improved quality of care (Bradford et al., 2024; Budd et al., 2022; Chomienne et al., 2011; Dath et al., 2014; Hannah et al., 2012; Johanson, 2021; Knowles et al., 2015; Miller‐Matero et al., 2016; Ross et al., 2019; Torrence et al., 2014; Vickers et al., 2013) and could increase continuity of care (Knowles et al., 2013). GPs also noticed a reduction in their mental health prescribing: ‘… my immediate feeling is that mine will be a fraction of where it was’ (Monnickendam et al., 2025). Psychology provision could also lead to positive changes in attendance rates (Lorentzatou et al., 2021) and improved communication between primary care and secondary mental health services (Dath et al., 2014). Additionally, doctors reported improved workplace well‐being, office atmosphere and quality of life at work (Chomienne et al., 2011; Monnickendam et al., 2025).

There was also a positive impact on workload. Staff reported that it has potential to reduce workload, free up staff time, make the workload more manageable (Austin, 2012; Chomienne et al., 2011; Farmanova et al., 2017; Hannah et al., 2012; Hartley et al., 2022; Monnickendam et al., 2025; Raybould, 2019) and improve HCP efficiency (Torrence et al., 2014). In turn, primary care staff reported a potential improvement in well‐being (Hannah et al., 2012; Monnickendam et al., 2025) and a reduction in personal stress levels (Farmanova et al., 2017; Miller‐Matero et al., 2016) and emotional burden/exhaustion (English et al., 2024; Hartley et al., 2022). Staff felt integration helped prevent them from feeling overwhelmed and burned out (Jewiss et al., 2023).

Educational and training opportunities appeared to be produced (Bradford et al., 2024; Hartley et al., 2022; Monnickendam et al., 2025). For example, psychology professionals could play an important role in staff development (Austin, 2012). There were increased opportunities for information sharing (Knowles et al., 2015), and psychologists could provide knowledge transfer sessions (Farmanova et al., 2017). Over time, this could build professional knowledge and skills (Jewiss et al., 2023), and staff described learning new skills and treatment techniques (Raybould, 2019; Seierstad et al., 2017; Turgesen, 2010). Primary care staff reported feeling more competent, confident and comfortable identifying, discussing and dealing with mental health problems in primary care (Austin, 2012; Bradford et al., 2024; Farmanova et al., 2017; Johanson, 2021; Knowles et al., 2013; Monnickendam et al., 2025; Torrence et al., 2014; Vogel et al., 2012). Further, HCPs described how this can enhance their confidence and ability to manage mood problems in the context of complex physical symptoms (Knowles et al., 2015).

#### Psychology professional perspective

Psychology professionals seemed satisfied working in primary care (Berkel et al., 2019; Kaitz & Ray, 2021). The role could be flexible and varied, and they valued collaboration and teamwork with physicians and patients (Berkel et al., 2019; Vogel et al., 2012).

[The primary care setting is] really dynamic …. It allows for creativity and flexible thinking …. You could be in a completely different situation every hour. (Berkel et al., 2019)

They also valued opportunities to learn from other professionals and being able to teach others about behavioural health and therapy (Berkel et al., 2019).

##### Challenging aspects of the role

There were potential challenges working in primary care. For example, some psychology professionals described working in these settings as emotionally exhausting and taxing, and anxiety provoking (Barajas et al., 2021; Berkel et al., 2019), with a quicker and more intense pace of work (Enos, 2016; Farmanova et al., 2017; Vogel et al., 2012) and higher caseloads (Farmanova et al., 2017). Time constraints could be challenging (Barajas et al., 2021; Berkel et al., 2019). One study described how initially up to 15 patients were booked into the psychologist clinic each day for 20‐ to 30‐min consultations; however, these were reduced to a maximum of 12 a day to slow down and to improve assessment skills (Hartley et al., 2022). Conversely, some psychologists felt underutilised (Berkel et al., 2019). In one study, the psychologists were dissatisfied with the level of administrative support (Kaitz & Ray, 2021).

There may also be challenges around role confusion (Barajas et al., 2021; Bradford et al., 2024); for example, there may be a lack of understanding of the role/responsibilities of psychology professionals (Budd et al., 2022), and they can be seen as only having expertise in mental health, not behavioural health (Berkel et al., 2019). Further, the model of care needs to be made clear to all members of the team (Nguyen et al., 2024). Other primary care staff may misunderstand the nuances of professional titles—one study with counselling psychologists discussed ‘one of my doctors introduces me as a clinical psychologist every single time he introduces me’ (Berkel et al., 2019). In terms of role boundaries, one study described that in early stages of integration, psychologists were concerned that physicians might be taking on cases that could be referred to them (Farmanova et al., 2017), whereas in another study, the psychology professional and nurses maintained their physical and mental health role boundaries (which was a barrier to collaborative care) (Knowles et al., 2013).

Some studies described primary care staff being resistant to integration (Farb et al., 2018); for example, primary care clinicians were sometimes reluctant to change their practices or were unaware how behavioural health services could be effective for improving health behaviours (Staab et al., 2022). There was also a reported poor sense of fit within some teams, with other team members not fully understanding how a psychologist can benefit the team (Berkel et al., 2019). Some psychology professionals reported feeling not fully integrated into the practice team (Budd et al., 2022).

##### Preparation for the role and training needs

Prior training in medical and integrated settings may support preparation for psychology professionals in the role (Berkel et al., 2019; De Master, 2011). One study described that internships focused on integrated health; coursework in primary care; mental health integration and interdisciplinary work; and continuing education (including primary care behavioural health certification) prepared psychology professionals for working in these settings (Berkel et al., 2019). Psychologists agreed that training in integrated primary care was valuable, regardless of whether they intended to eventually practise in an integrated setting, and most valued was the ability to streamline the assessment/triage process, to gain knowledge about a wide variety of evidence‐based interventions delivered in a brief timeframe and to develop skills in collaborating with physicians (Vogel et al., 2012). Direct support from supervising psychologists may assist psychologists in transitioning from traditional mental health practice to integrated primary care (Vogel et al., 2012).

Some behavioural health clinicians may lack training in integrated care or find it difficult to adhere to brief interventions while serving high‐need patients (Nguyen et al., 2024; Staab et al., 2022), and interestingly, in one study, both GPs and psychology specialists stated that not everyone may be comfortable or do a good job in such a setting (Seierstad et al., 2017). Finally, in a UK study, it was reported that 95% of consultations were with the senior clinical psychologist (Durcan, 2020) (as opposed to training grades), reflecting the level of experience needed by behavioural health experts working in primary care.

#### Working together

Co‐location can allow for informal collaboration (Knowles et al., 2013), and close collaboration may facilitate both optimal care and continuity of care (Hermens et al., 2014; Miller et al., 2023). Increased collaboration can result in both professional groups feeling more comfortable working together, with mutual respect and trust evolving (Farb et al., 2018). Success of interprofessional collaboration and integrated care delivery may vary (Hermens et al., 2014; Knowles et al., 2013) from poor understanding: ‘GP's don't even, I don't think they know what the service does, never mind the role of the practitioner’ (Knowles et al., 2013) to collaboration: ‘Last week there was a question that I had about somebody's diabetes and the use of insulin … I actually just popped my head around the door for one of the practice nurses … just being there meant that I could ask her that’ (Knowles et al., 2013).

One study discussed how integrated working defaulted to be between the psychology professionals and practice nurses, with GPs having little involvement (Knowles et al., 2015). It may be that some physicians maximise opportunities to engage with behavioural health providers and offer integrated care, whereas others take a minimalist approach (Jewiss et al., 2023). One study described that interprofessional hierarchies could be a challenge, where power differentials could reduce the effectiveness of behavioural health integration (Malâtre‐Lansac et al., 2020).

Communication can be key, and regular communication between different care providers can facilitate high quality care (Gidding et al., 2014) and may be crucial to successful collaboration (Reid et al., 2021).

#### Professional considerations

Primary care staff suggest that there should be increased integration and support from psychology professionals in primary care (Austin, 2012; Bradford et al., 2024; Chomienne et al., 2011; Turgesen, 2010). Further, there were discussions that primary mental health care could be strengthened (Hermens et al., 2014) and that better cross‐sectoral collaboration could be developed (Reid et al., 2021). Staff also suggested that behavioural health and primary care staff should provide training for each other (Farb et al., 2018).

Psychology professionals recommended a role in primary care to psychology graduates (Budd et al., 2022). Psychologists working in these settings emphasise that assertiveness, flexibility and the use of brief interventions are likely necessary skills, in addition to individual and group therapy, supervision and assessment (Barajas et al., 2021). This was supported by findings that suggest that psychologists may need to develop resources and skills that better fit the brief service delivery model (Vogel et al., 2012). In addition, supervision is a necessary skill (Barajas et al., 2021) and is vital for therapists working in primary care (Howard, 2012). Also, there is a need for education and liaison with GPs regarding the services psychology professionals can offer (Bassilios et al., 2014). In terms of care delivery, one study described how care plans needed to integrate biopsychosocial elements as appropriate to the complexity of service user presentations (Kierans & Byrne, 2010).

There were also recommendations for communication and collaboration. One study highlighted key qualities that facilitate good collaboration, including professionals being comfortable with each other (described as particularly important when psychologists are co‐located in primary care teams); confidence in each other's professional competence (perceived as a prerequisite for an effective referral system); and mutual respect (Farmanova et al., 2017). Further, any procedures/activities (e.g. scheduled meetings and co‐provision of care) related to more formal types of collaborations should be set out clearly before collaborative processes begin.

### Organisations

#### Information sharing and communication

Patients suggested that increased and shared access to patient records was a positive move and one that fitted within the holistic model of care (Berry, 2020).

I suppose that in a way it's good for them to understand what's actually happening, mentally or psychologically, to … better prescribe or better help … and that's actually probably quite good to know what your GP will be able to check in on your notes, see where you're at. (Berry, 2020)

Communication between primary care staff and psychology professionals may be facilitated by various methods:

- physical proximity (co‐location of a psychology professional) (Austin, 2012; Jewiss et al., 2023; Vickers et al., 2013);
- morning huddles (Jewiss et al., 2023; Nguyen et al., 2024);
- the electronic health record (EHR) (Austin, 2012; Cos et al., 2022; Ellbin et al., 2024; Farmanova et al., 2017; Jewiss et al., 2023), where doctors rated the quality of feedback from the psychologists' notes as excellent (Chomienne et al., 2011);
- chat applications, group texts, emails and telephone (Austin, 2012; Bradford et al., 2024; Cos et al., 2022; Jewiss et al., 2023; Miller et al., 2023; Nguyen et al., 2024);
- open door policy/knocking on doors (Austin, 2012; Ellbin et al., 2024; Jewiss et al., 2023);
- ‘corridor consultations’ (Austin, 2012; Farmanova et al., 2017); and
- warm handoffs (a formal introduction to a behavioural health clinician by another HCP) (Dueweke, 2019; Farb et al., 2018; Jewiss et al., 2023; Vickers et al., 2013), which facilitated appointment attendance as a patient in one study stated that without the warm handoff, they would not have attended their recommended behavioural health appointments (Dueweke, 2019).

#### Referrals

There were reports of GPs and nurses having different understandings of the referral criteria to the co‐located psychologist (Fletcher, 2021). According to psychologists, the reason behind the referral from physicians was not always clear (Ellbin et al., 2024). Primary care providers identified that a lack of time during appointments was a barrier to referring patients (Miller‐Matero et al., 2019). There was also reluctance from GPs to make a referral to psychology (IAPT service) even when patients asked to be referred (Yasmin‐Qureshi & Ledwith, 2020).

I went to the doctor and he told me but I feel he was very reluctant even when he did tell me [about the psychology provision]. (Yasmin‐Qureshi & Ledwith, 2020)

Further, psychologists felt that because GPs sometimes wait too long before referring and because of long waiting lists, patient symptoms worsen unnecessarily before they receive care (Gidding et al., 2014). One study found that less than half (41%) of those who received a referral attended services (Brooks et al., 2016).

There is potential for onward referrals to outside services/secondary care to be fewer and more appropriate with a co‐located/integrated psychology professional. GPs suggested improvements in their understanding of different services, with the psychologist acting as a ‘pathway navigator’ (Monnickendam et al., 2025). The presence of behavioural health specialists may give physicians and patients a preferable alternative to outside referrals, for which a patient likely would not be able to see a provider for a couple of months (Enos, 2016). Primary care providers noted they were making fewer referrals to outside providers—referrals were still used, but they were for reasons such as patient preference or need for help with a specific disorder (e.g. eating disorders and obsessive‐compulsive disorder) rather than general lack of access (Vickers et al., 2013). A clinical psychologist in one study described how onward referrals were only made when necessary: ‘… the vast majority of referrals that we make are accepted and “stick” with the service we refer to ….’ This was due to the detailed work by the psychologist ensuring the case was appropriate for referral and preparing the patient on what to expect from secondary care (or other) services, so they were more accepting of any offer made to them, described as ‘psychological readiness’ (Durcan, 2020). Physicians were also more likely to request feedback from the psychologist to whom they referred a patient in the community (Farmanova et al., 2017).

#### Organisational challenges

##### Access and appointments

Staff and patients highlight importance of not placing restrictions on service access, and they value rapid access to appointments (Raybould, 2019). Some studies reported that there was limited access to the psychology professional (e.g. due to staff shortages and long waitlists) (McElvaney & Timulak, 2013; Miller et al., 2023). Some organisations arranged blocks of time where the psychology professional is ‘on call’ (rather than in a scheduled session) to maximise their availability (Jewiss et al., 2023). Issues were raised around only providing appointments during working hours/not offering out‐of‐hours appointments (Dath et al., 2014; Hard et al., 2015). Further, some studies reported long waits from first/assessment appointment to subsequent appointments (Berry, 2020; Yasmin‐Qureshi & Ledwith, 2020). One study described recruitment and retention of behavioural health consultants as a challenge (Klege et al., 2025), and another study explained that challenges resulted from limited behavioural health staffing levels that impeded patients' timely access to services (Jewiss et al., 2023).

It was suggested that appointment numbers were limited and the appointment time allocated was too brief (Dueweke, 2019). One study described that behavioural health clinicians (who may be accustomed to 50‐min appointments and long‐term patient relationships) could have challenges acculturating to primary care (Malâtre‐Lansac et al., 2020). This was especially true for collaborative care models that featured brief patient interactions and focused on mild‐to‐moderate needs (with severe needs referred onwards) (Malâtre‐Lansac et al., 2020). Finally, there were mixed opinions from patients on the acceptability of group interventions/appointments (Arfuch et al., 2021; Arfuch et al., 2022).

##### Physical environment and equipment

There were suggestions of physical space challenges, for example, finding space due to limitations of the building (Nguyen et al., 2024); additionally, though there is value in co‐location, some studies suggested a preference for discussing mental health problems in a separate space from the nurse or physician (Knowles et al., 2015; Miesner, 2014). From the psychology professional's perspective, there were issues around not being provided with a room (Budd et al., 2022). Further, being co‐located, but in a different part of the building, can stifle collaborative working (Ellbin et al., 2024; Nguyen et al., 2024). There can also be issues with limited access to practice information systems (Knowles et al., 2013), and some EHRs are ‘not designed for behavioural health integration’ (Malâtre‐Lansac et al., 2020).

#### Organisational considerations

##### Information sharing and communication

Communication was discussed as a prerequisite for good collaboration between different HCPs (Ellbin et al., 2024). Psychology professionals and primary care staff suggested that increased communication may be helpful to resolve issues (e.g. unclear roles and practical issues like finding room space) (Budd et al., 2022). In terms of warm handoffs, good communication and effective handoffs between primary care and behavioural health can be crucial (Enos, 2016), and staff stressed the need for the person doing the handoff to clearly explain the behavioural health provider's role to patients (Jewiss et al., 2023; Nguyen et al., 2024).

It was discussed that communication should take place via the EHR (Gidding et al., 2014). Further, there was an expressed need for more integrated health information technology, particularly behavioural health documentation templates (Staab et al., 2022). Therefore, it can be important that user‐friendly systems for collaborating on patient care and determining when behavioural health providers are available are provided (Jewiss et al., 2023).

##### Referrals

One study described how regular feedback from the psychologists to the medical providers regarding referrals would be of benefit (e.g. regarding correct referrals) (Fletcher, 2021). Additionally, it was suggested that physicians making referrals should follow up with patients who stop sessions with the psychologist to find out the reason (Farmanova et al., 2017).

##### Appointments

A scheduling system to connect the psychology professional can be useful to ensure patients who could benefit from behavioural health are not missed (Nguyen et al., 2024). Patients highlight a preference for consistency of care (e.g. only seeing one psychology professional) (Berry, 2020; Gomez, 2017). Both patients and staff appear to want more sessions (Budd et al., 2022). Appointments could benefit from being flexible (e.g. frequent appointments, evening hour options and more immediate access) (Farmanova et al., 2017; Miller et al., 2023; Seierstad et al., 2017). One study found that in‐person and bi‐weekly sessions may be preferred (Miller‐Matero et al., 2019), and there were suggestions to include families/carers in appointments (Arfuch et al., 2022; Miller‐Matero et al., 2019). Further, sufficient investment in staff is valued, so that new patients can access services in a timely manner (Jewiss et al., 2023).

## DISCUSSION

This scoping review aimed to explore how psychology professionals have been co‐located/integrated in primary care to synthesise the views and experiences of patients, primary care staff and psychology professional groups on this role. It aimed to identify areas for research, prior to the development of an implementation strategy. A total of 72 sources were included, and it appeared that patients, primary care staff and psychology professional groups described the provision of psychology in primary care as positive. The study characteristics and results aligned to a prior systematic review (exploring implementation of integrated primary care in the United States), which found variation in professional types (e.g. psychologists, counsellors and behavioural health consultants) and variations in interventions delivered in these settings (e.g. CBT, psychoeducation etc.) (Martin et al., 2014). Our review shows that psychology professionals in primary care have the potential to support patients with a wide range of health conditions.

Having a co‐located/integrated psychology professional can lead to patient satisfaction, increased and/or improved access to care for patients and reduced stigma. This is an important finding as it aligns with policies to reduce health inequalities (e.g. United Nations [UN] Sustainable Development Goals [UN, 2024], NHS long‐term plan [NHS, 2019] and NHS Health Scotland health inequalities policy [NHS Health Scotland, 2013]). Further, the provision has the potential to improve patient knowledge/skills, and in a review of over 60 years of research, it has been found that patient education positively affects health outcomes (e.g. for diabetes, respiratory conditions [e.g. asthma and COPD] and mental health problems) (Simonsmeier et al., 2022).

Primary care staff seemed satisfied with having a psychology professional, and staff felt more competent, confident and comfortable identifying, discussing and dealing with mental health problems. A recent review found that integrated care promotes interprofessional collaborations and teamwork, supporting job satisfaction for primary HCPs (Liu et al., 2023). This same review found mixed results for work stress of primary HCPs, with some reporting more burnout due to increased workload of integrated care and some reporting that expanding roles reduced work stress. It should be noted that this review included a wide range of professionals under the umbrella of integrated care (e.g. public health, mental health and pharmacy) and was not specific to psychology. Critically, the current scoping review found that psychology provision has potential to reduce primary care staff workload, make the workload more manageable and reduce personal stress levels of clinicians. Work amount and intensity are among the most common reasons for GPs intending to leave the profession sooner than planned (Owen et al., 2019). Further, higher GP workload is associated with increased burnout levels, and burnout may lead to negative impacts on patient safety (Hall et al., 2019). Therefore, the provision of psychology professionals in primary care could improve the GP retention crisis and patient safety.

The current scoping review highlights that psychology professional groups describe being satisfied working in primary care. They find the role flexible and varied, and they value collaboration and teamwork with physicians and patients. Further, they value opportunities to learn from other professionals and be able to teach others about behavioural health and therapy. However, the review found that the role is not without its challenges. Psychology professionals report that the role can be limited by time constraints and be emotionally demanding and that the pace of work can be intense. They also report a perceived lack of understanding from primary care staff of the role/responsibilities of psychology professionals in primary care, and some staff can be resistant to integration, which could lead to a poor sense of fit within teams. This highlights a need to develop research in this area that addresses the challenges and produces an optimal model of care prior to routine implementation.

In addition, prior to routine implementation of psychology provision in UK primary care practices, the current review highlights a need to explore the specific skills and training required for psychology professionals working in these settings. For example, patients were dissatisfied with inflexible, manualised approaches (Yasmin‐Qureshi & Ledwith, 2020) and acknowledged limitations in support that T/APPs (NHS Band 4) were able to provide (Budd et al., 2022), which suggests that professionals should be trained to a level where they can provide flexible/tailored interventions (e.g. doctoral level). This was reflected in one study, where the senior psychologist (as opposed to training grades) was delivering almost all of the consultations (Durcan, 2020), highlighting the potential level of expertise needed. Additionally, the review found that prior training in medical and integrated settings may prepare psychology professional groups for the role (Berkel et al., 2019; De Master, 2011) and that those with a lack of training in this area may experience challenges (Staab et al., 2022). Research also identified that both GP and psychology groups felt that not everyone would be comfortable working in primary care (Seierstad et al., 2017). This emphasises a need to develop research in this area to produce clear training guidelines, such that psychology professionals are prepared for the role prior to co‐location/integration into the primary care team.

### Future research

Although implementation considerations have been identified, this scoping review highlights the need to develop research in this area. The review suggests that psychology provision in primary care is likely acceptable to stakeholders (patients, primary care staff and psychology professionals). However, most included sources were from outside of the United Kingdom, with different health systems, and sources included a wide range in the type/volume of psychology provision. This shows that ahead of implementation, further research investigating an optimal model of care in a UK general practice context is required. As previously mentioned, research into the training needs of co‐located/integrated psychology professionals is vital to inform UK psychology training programmes, with a view to providing the necessary skills for a future workforce. Further, an evaluation of psychologists currently working in UK primary care should be conducted to explore barriers and facilitators to employing psychologists (Hart et al., 2023), to understand optimal ways of working and to develop implementation and service delivery guidelines. Finally, as suggested by Lepièce et al. (2023), research on collaboration between GPs and psychologists should be conducted to identify efficient collaborative strategies and to assess the impact of interprofessional collaboration on patient health outcomes.

### Strengths and limitations

A strength of this review is that (to the authors' best knowledge) this is the first review that summarises available evidence on stakeholder views of psychology provision in primary care. A further strength is that the categories were synthesised in discussion with the multidisciplinary authors (health psychologists and GPs) who have worked in primary care. There are however limitations. Due to feasibility and for practical reasons, our searches were time limited (e.g. 2010–present) and restricted to four electronic databases. Further, search terms pertaining to views and experiences were not included; however, this was to ensure that stakeholder views were captured across all study designs. Additionally, as psychologists are not routinely embedded in UK primary care, the review included a wide range of psychology professional groups (including trainees) from international contexts. Although studies that could not be applied to a UK context were excluded, this range of professional groups may affect the conclusions of the review. It was anticipated that a wider range of quality improvement initiatives and clinical pilot studies where psychology professionals were co‐located/integrated into primary care would have been sourced for the current review. Following scoping review methodology, no quality assessment was applied (Grant & Booth, 2009), this places limits on conclusions about effectiveness, though this does not detract from our narrative description of patient and professional views. Finally, although we searched studies internationally, only studies applied to UK primary care practices were included, which limits the generalisability of the findings.

## CONCLUSIONS

This review finds that the views and experiences of patients and professionals on psychology professional provision in primary care are broadly positive. However, the co‐location/integration of psychology professional groups in primary care is not without its challenges. Considerations prior to implementation have been generated from this review (e.g. suitability of patients and interprofessional communication facilitators), and clear further research areas have been identified (e.g. training needs). This review provides the foundation for further research, which is needed prior to routine implementation of psychologists in UK primary care.

## ETHICS STATEMENT

The authors have nothing to report.

## CONFLICT OF INTEREST STATEMENT

The authors declare no potential conflicts of interest with respect to the research, authorship and/or publication of this article.

## Supporting information


**Data S1.** Supporting Information

**Data S2.** Supporting Information

## Data Availability

The authors confirm that the data supporting the findings of this review are available within the article and its supporting information.
