# Supplementary material for: Psychologists in primary care: A scoping review exploring the views and experiences of patients and professionals on psychology provision in primary care
Source: Appl Psychol Health Well Being. 2026 Jul 9;18(4):e70178. doi: 10.1111/aphw.70178 (PMC13348508; doi:10.1111/aphw.70178)
Supplement: Supplementary file 1 — Data S1. Supporting Information [file APHW-18-0-s001.docx]

**Supplementary File 1**

| **PsycINFO (Ovid platform) (19-06-23)**  **Records identified:** 4385 | |
| --- | --- |
| 1 | psycholog*.tw. |
| 2 | “Behavioural health".tw. |
| 3 | "Behavioral health".tw. |
| 4 | "Primary care".tw. |
| 5 | "Primary health care".tw. |
| 6 | "Family medicine".tw. |
| 7 | "General practice".tw. |
| 8 | "Community health".tw. |
| 9 | 1 or 2 or 3 |
| 10 | 4 or 5 or 6 or 7 or 8 |
| 11 | limit 10 to (english language and yr="2010 -Current") |
| 12 | 9 and 10 |
| 13 | 11 and 12 |
