# Supplementary material for: Psychologists in primary care: A scoping review exploring the views and experiences of patients and professionals on psychology provision in primary care
Source: Appl Psychol Health Well Being. 2026 Jul 9;18(4):e70178. doi: 10.1111/aphw.70178 (PMC13348508; doi:10.1111/aphw.70178)
Supplement: Supplementary file 2 — Data S2. Supporting Information [file APHW-18-0-s002.pdf]

Supplementary File 2

No.	Study (year)/ Setting/ Country	Research Aim(s)	Condition(s)	Psychology Professional Group	Service/intervention(s) delivered	Research Methods	Participants	Key findings (relevant to views/experiences of patients and/or professionals on psychology provision in primary care)
1.	Allen et al. (2025)<br><br>Primary care clinics (n=2)<br><br>USA	To obtain qualitative feedback from primary care behavioural health (PCBH) patients about the benefits of PCBH services.	Various (e.g. anxiety, depression, trauma and stress related disorders, sleep etc.)	Behavioural health interns/ consultants	• Various psychological approaches (e.g. CBT techniques, goal settings, psychoeducation, resource sharing etc.).	Qualitative (survey)	Patients (n=135)	• Patients felt they were given a space to talk, they were validated by the BHC (through listening, reassuring, and validating the patient's experience), and there was instillation of hope (the BHC expressing a commitment to both the patient and their progress beyond a single meeting).<br>*Helpful psychological intervention approaches:*<br>• Patients found resource sharing helpful (e.g. the BHC utilising websites, handouts, apps, or other media). A unique aspect of resource sharing occasionally required the BHC to serve as a healthcare team liaison, facilitating communication with other members of the team based on patient concerns (e.g. upping medication dosage).<br>• Goal setting and treatment planning was impactful e.g. discussing and outlining clear, measurable objectives toward which the patient could work.<br>• Skill building was an important component of BH services, defined as the BHC teaching the patient a particular skill (e.g., how to relax their muscles).<br>• Psychoeducation or guidance was commonly used, involving the BHC providing information to patients on thoughts, beliefs, emotions, or behaviour.<br>• Specific CBT techniques were also reported by patients. One participant commented that he found the *"record keeping"* to be helpful.
2.	Angantyr et al. (2015)<br><br>Primary care centres (n=3)<br><br>Sweden	Examine experiences of the primary care behavioural health model at primary care centres in Sweden, by studying the effects of symptoms and functioning; participant satisfaction and factors affecting satisfaction, and gender differences regarding effects and satisfaction with the psychology consultation.	Various (e.g. anxiety, stress, depressed mood, sleep problems, pain, and others)	Psychology consultant	• Initial 30-minute appointment, followed by additional 15–20-minute follow-up sessions (if deemed necessary).<br>• Psychoeducational interventions and action for behavioural change.	Quantitative (questionnaire)	Patients (n=54)	• Participants generally felt a high degree of satisfaction. The mean score for the client satisfaction questionnaire in the study was 22.62 (SD = 6.08, range = 10-32), with the greater number of points indicating a greater degree of satisfaction.
3.	Arfuch et al. (2022)<br><br>Primary care centres (n=5)<br><br>Spain	To understand patients' lived experiences during a proposed multicomponent intervention (MCI) program for fibromyalgia syndrome.	Fibromyalgia syndrome	Psychologist	• The MCI program includes a 12-week/2-hour session group-based interdisciplinary program.<br>• The program combines health education, physical exercise and cognitive behavioural therapy (CBT).<br>• Topics included: neurophysiology, pharmacology	Qualitative (interviews)	Patients (n=10)	• Participants agreed that the program facilitated illness knowledge and acceptance, and that it improved coping skills and symptom self-management in daily life.<br>• Participants discussed benefits of the group setting leading to new encounters, identification processes, and social networks. The group modality offered the opportunity to create new social networking with alike people who were facing similar challenges. However, the group setting was also found to be a barrier (e.g. members using the group as a shield, and negative impacts of social comparisons).

No.	Study (year)/ Setting/ Country	Research Aim(s)	Condition(s)	Psychology Professional Group	Service/intervention(s) delivered	Research Methods	Participants	Key findings (relevant to views/experiences of patients and/or professionals on psychology provision in primary care)
					of pain, postural hygiene, nutrition, insomnia management, memory, sexuality, breathing and relaxation techniques, stretching, exercises, emotional management.			• Suggestions for improvement included more psychological guidance, expressive group therapy, and inviting family members to sessions to help them to understand the diagnosis/consequences.
4.	Arfuch et al. (2021)<br><br>Primary care centres (n=11)<br><br>Spain	To assess patients' experiences and appraisals about a complex intervention programme for fibromyalgia syndrome in primary care centres.	Fibromyalgia syndrome	Psychologist	• The intervention included 12-week/2-hour session group-based programme combining: education, physical exercise, and CBT based on pain and attention management, learning to manage emotions, strategies for coping with difficulty, and pleasurable activities planning.<br>• This programme aims to promote patients' literacy and skills development for fibromyalgia management, enhance their physical status, and reduce their emotional distress in order to overcome psychological difficulties.	Qualitative (focus groups)	Patients (n=19)	• Patients were satisfied with the intervention.<br>• Patients agreed that there was a positive impact of the intervention on their quality of life. Although they did not report significant changes in physical symptoms (e.g. pain). The programme allowed them to improve their lifestyles by: incorporating healthy habits and routines, reducing their pharmacological intake, developing a positive attitude towards pain, and enriching their psychological and social well-being.<br>• Participants highlighted the coping skills acquired during the programme, and emphasised the acquisition of self-understanding, self-control, and self-management of the syndrome.<br>• The programme was acknowledged for its educational and health benefits as well as for its positive group effect.<br>• Regarding the group approach, participants showed acceptance, acknowledging its benefits but also remarking its drawbacks e.g. participants proposed including the family.<br>• Patients were satisfied with the professionals.<br>• Participants claimed that the programme timeframe was not sufficiently long enough to cover all its contents in-depth and offer them time to work on their suffering.
5.	Austin (2012)<br><br>Integrated Primary Care (IPC) clinics that provide IPC for rural and underserved patients (n=3, 2 family medical practices, 1 federally qualified health centre)<br><br>USA	To explore how rural physicians in integrated primary care settings experience integrated primary care.	Behavioural and mental health	Clinic 1: Behavioural health consultant (BHC) (doctoral level psychologist) (n=1), doctoral level psychologist (n=1)<br><br>Clinic 2: Clinical psychologist (who serve as BHCs) (n=3)<br><br>Clinical 3: Mental health counsellor (n=1)	• Behavioural and mental health interventions.<br>• Delivered directly in the clinics.	Qualitative (interviews)	Primary care physicians (medical doctors, doctors of osteopathic medicine) (n=7)	• Ongoing patient evaluation and monitoring by BHCs provided physicians with diagnostic clarity and confidence. Physicians felt the extended time available to the BHCs, as well as their enhanced skill at conducting psychosocial interviews, promoted more thorough understanding of patients. Having BHCs onsite may have destigmatised patient's experience of receiving mental health treatment, which may improve the quality of the physician-patient relationship.<br>• BHCs have helped patients access specialty mental health care. Physicians appreciated how convenient access to expert consultation from BHCs supported them in working with challenging patients. Physicians appreciated being able to make in-house referrals for targeted behavioural health care (e.g. chronic disease management, health promotion). Physicians talked about the importance of physical proximity to the BHC's office, in combination with the BHC's open door policy, in permitting the *"curbside"* consultations that are critical given the unpredictable nature of primary care. Most of the physicians spoke of desiring more psychiatric support.<br>• Each participant described benefits that BHCs offered in directly engaging challenging patients and in assuming responsibility for time-consuming aspects of their work, which allows physicians to work more productively and efficiently. BHCs played an important role in supporting and developing primary care staff at professional and personal levels.

No.	Study (year)/ Setting/ Country	Research Aim(s)	Condition(s)	Psychology Professional Group	Service/intervention(s) delivered	Research Methods	Participants	Key findings (relevant to views/experiences of patients and/or professionals on psychology provision in primary care)
								This team development and support was crucial for efficient handling of BH conditions. Participants spoke of the importance of ongoing in-house training of managing mental health in primary care.<br>• One physician said her site's model of integrated primary care was not satisfactory, as it offered little in the way of consultation or timely assistance with complex patients. IPC at this clinic was also hampered by a tedious BHC referral process for each patient. There was also a mismatch between the specialised expertise desired by the physician, and the areas of expertise of her BHC.
6.	Barajas et al. (2021)<br><br>Integrated behavioural health (IBH) settings<br><br>Not reported	To explore models of IBH with a focus on the principles, training, and experiences of social workers and psychologists working in IBH settings.	Not reported	Psychologist	• Psychologists working in IBH settings.	Literature review	Psychologists (n=not reported)<br><br>Social workers (n=not reported)	• Psychologists working in IBH settings emphasised individual and group therapy, supervision and assessment, assertiveness, flexibility, and use of brief interventions as necessary skills.<br>• Psychologists also report patient psychoeducation and providing culturally relevant care as key aspects of their professional roles.<br>• Time constraints, emotional exhaustion, and role confusion were among the most commonly cited challenges by psychologists working in IBH.
7.	Bassilios et al. (2014)<br><br>Divisions of General Practice (n=22)<br><br>Australia	To elicit lessons learnt from the implementation of a telephone-based CBT pilot.	High prevalence disorders (e.g. depression and anxiety)	Mental health professional (equipped to deliver CBT by telephone)	• Telephone-based CBT (T-CBT) pilot, which involved substituting or combining T-CBT with face-to-face services.<br>• Delivering behavioural and cognitive interventions, 1h in duration.<br>• Unclear where sessions were delivered, but the therapy allowed for services to be delivered in rooms at the general practices.	Qualitative (interviews)	Access to Allied Psychological Services project officers (n=22)<br><br>Mental health professionals (6 psychologists, 2 social workers, 1 psychiatric nurse, 1 psychotherapist/ sociologist/coun sellor) (n=10)	• Project officers stated that positive provider responses facilitated the T-CBT pilot: *"excellent clinicians who are enthusiastic and GPs who are open minded"*, and the need for counselling services to be provided remotely was reported to facilitate the pilot.<br>• The increase in accessibility for consumers that may otherwise experience difficulties accessing psychological services (including those living in rural or remote areas), was the most frequently mentioned positive impact of the T-CBT pilot. The ability to offer high-need consumers telephone contact was also perceived positively.<br>• The most commonly mentioned barrier to T-CBT was the low rate of GP referrals, which was attributed to difficulty convincing GPs to use the service, which in turn was probably attributable to their (and consumers') preference for face-to-face treatment.<br>• The most common suggestion for improvement of the pilot made by mental health professionals, was the need for increased education and liaison with GPs regarding the services (GPs were not aware of the program).
8.	Beacham et al. (2012)<br><br>Federally Qualified Health Centre (FQHC) (n=5, with access to onsite/ integrated behavioural health services)	To examine attitudes regarding behavioural health services among primary care medical providers in practices that offer such services via onsite behavioural health providers and practices that do not.	Mental health and behavioural medicine issues	Advanced clinical psychology doctoral student	• Access to onsite/integrated behavioural health services delivered by licensed clinical social workers or advanced clinical psychology doctoral students at the clinic a minimum of 1 day per week.	Quantitative and qualitative (survey)	Physicians (n=16) and nurses (n=15) with access to onsite/ integrated behavioural health services<br><br>Physicians (n=62) with no	• The sample with access to behavioural health services agreed more overall on knowledge/attitudes on a 'biopsychosocial conceptualisation subscale' (e.g. *"Patient's physical health and recovery from illness is influenced significantly by their emotional health and level of social support available to them"*) compared to the sample with no access.<br>• The sample with access to behavioural health services agreed more on an 'importance of mental health provider subscale' (e.g. "*I frequently use behavioural medicine specialists to support my patients' lifestyle changes (i.e., smoking, weight loss)"*), and on an 'access to mental health/behavioural medicine services subscale' (e.g. *"My patients have easy access to a behavioural medicine expert (i.e. chronic pain management, sleep hygiene, medication adherence issues)"*).

No.	Study (year)/ Setting/ Country	Research Aim(s)	Condition(s)	Psychology Professional Group	Service/intervention(s) delivered	Research Methods	Participants	Key findings (relevant to views/experiences of patients and/or professionals on psychology provision in primary care)
	Family medicine practices and women's health physician practices (n=not reported, with no access to onsite behavioural health services)<br><br>USA						access to onsite behavioural health services	• The sample with access to behavioural health services reported a greater need of behavioural health services, further they reported higher attempt to involve mental health or behavioural medicine colleagues compared to the sample with no access.
9.	Berkel et al. (2019)<br><br>Family practice clinic (n=1), weight loss centre (n=1), home-based primary care through Veterans Affairs (n=1), university health centre (n=1)<br><br>USA	To explore the experiences of early career counselling psychologists and the roles they play in integrated primary care.	Various mental health and chronic health conditions (e.g. anxiety, depression, diabetes, obesity, sleep problems, substance abuse)	Early career counselling psychologist	• Collaborating and consulting.<br>• Assessments using a variety of screening tools and therapeutic interventions (including individual and group counselling).<br>• Conducting supervision and teaching.<br>• Varied responsibilities including research, prevention work, and administrative tasks.	Qualitative (interviews)	Early career counselling psychologists (PhD in counselling psychology) (n=13)	• Training participants described they received to prepare them for working in integrated health care settings included: practical (numerous practical work) in integrated health settings; pre-doctoral internships that focused on, or included rotations in, integrated health; post-doctoral fellowships and residencies; coursework in primary care, mental health integration, and interdisciplinary work; and continuing education, including Primary Care Behavioural Health certification).<br>• They saw the integrated role as flexible and varied.<br>• Participants were satisfied with working in integrated healthcare. Satisfaction was related to collaboration and teamwork, including appreciation of interprofessional collaboration and how it improved the quality of their work; providing good patient care; education and training, having the opportunity to learn from other professionals and being able to teach others about behavioural health and therapy; feeling appreciated. Psychologists reported interdisciplinary collaboration and communication as paramount to these settings, and that collaboration improved the quality of their work. They referred to their work settings as *"holistic"* and *"interdisciplinary"* and as providing better access and care to patients by incorporating the skills of multiple providers.<br>• Some participants talked about patients who were hesitant to see a psychologist when they were just planning to see their primary care physician, sometimes hearing, *"I'm not crazy, why do I have to see you?"* In response, participants make efforts to reduce confusion and stigma. In addition to teaching patients about their services, a few participants also shared that they train other providers about their role/services, because it is beneficial when team members can help clarify the role for patients.<br>• Participants had different professional titles, most common was Behavioural Health Consultant, four were Psychologist, one Consulting Psychologist, one Paediatric Psychologist, one Postdoctoral Fellow, and one Behavioural Health Psychology Resident. Several participants stated that their titles were not necessarily how others identified them at work e.g. one participant stated that she was referred to as a *"behaviourist"* by staff, and another said *"one of my doctors introduces me as a clinical psychologist every single time he introduces me."*

No.	Study (year)/ Setting/ Country	Research Aim(s)	Condition(s)	Psychology Professional Group	Service/intervention(s) delivered	Research Methods	Participants	Key findings (relevant to views/experiences of patients and/or professionals on psychology provision in primary care)
								• Challenges included, a general lack of understanding/under appreciation of their role as psychologists e.g. frustration that psychologists were often seen as only having expertise in mental health—not behavioural health—which could help with diabetes, asthma etc.; a poor sense of fit within the team - the feeling that other team members do not fully understand how the psychologist can benefit the team was common; time constraints; the impact of hierarchy; feeling underutilised; work that is emotionally taxing.
10.	Berry (2020)<br><br>Primary health organisation (n=1)<br><br>New Zealand	To investigate the experiences of patients who participated in the Focused Acceptance and Commitment Therapy (fACT) therapy service offered at a primary healthcare organisation in Palmerston North, New Zealand.	Various (psychological complaints/ conditions; health conditions e.g. diabetes, heart disease, cancer)	Psychologist (also referred to as Health Improvement Practitioner)	• fACT is conducted in a series of single sessions, where the patient is required to make changes and seek further support, if required.<br>• The therapy focuses on mindfulness techniques alongside following a values-based action plan for tackling unwanted or distressing thoughts.	Qualitative (interviews)	Patients (n=10)	• Patients appreciated a non-medication alternative.<br>• The experience of accessing mental health services from a location which was familiar and acceptable to them reduced stigma and was seen as positive.<br>• Many patients highlighted practical aspects of the service, such as its physical location, as positive. Some expressed the physical toll that *'traipsing around'* between doctors, specialists and other health professionals can have on someone who is already feeling demotivated, fatigued and not themselves.<br>• Delays in being able to access the service either from the first or subsequent appointments had a direct negative effect on each participant's overall experience.<br>• Participants with more serious conditions also spoke of feeling "rushed" in their appointments. Many believed they were 'owed' an hour.<br>• Overwhelmingly, the majority of participants believed that increased and shared access to notes was positive, and one which fitted within their holistic model of care. This allows the patient, GP, and other clinicians associated with the clinic to be able to access the notes of any consultations left by the clinician.<br>• The ability (or not) of participants to see the same psychologist to build a rapport and relationship with had a large effect on how they viewed their overall experience. For all participants who saw more than one psychologist, they reported this experience to be negative, and expressed feelings of loss, discomfort and annoyance with what they perceived to be a lack of momentum in their care.<br>• Patients were mixed in their reflections on the process on relationship building with the psychologist(s). Those with less serious conditions made faster and stronger bonds and those with more serious conditions were more critical of their therapist relationships.
11.	Bradford et al. (2024)<br><br>Federally Qualified Health Centre (FQHC) (n=3)<br><br>USA	To obtain qualitative feedback of staff perspectives and experiences following implementing a behavioural health integration (BHI) model into their FQHC practices.	Not reported	Behavioural health consultant (BHC)	• Behavioural Health Integration Model/Integration of Behavioural Health Consultants (e.g. standardised screening for depression, established workflow practice, interdisciplinary team-based approach, psychotherapy, consultation for psychiatric medication, patient-centred care and flexibility in Community Health Centre practices).	Qualitative (interviews)	Behavioural health consultants (n=8)<br><br>Nurse practitioner (n=6)<br><br>Physician (n=5)<br><br>Care manager (n=1)	*Barriers to behavioural health integration:*<br>• *Lack of access and availability of the BHC:* staff found it challenging to provide consistent care to patients when the BHC was unavailable (with another patient or no BHC scheduled that day), which created issues within the clinics' workflow/time delays/communication challenges. Staff desired an increase in BHC availability.<br>• *Inconsistent use of the behavioural care team:* need for increased education/training on how and when to involve the BHC in patient care. Staff confusion about how and when BHCs were involved led to confusion about roles. Primary care team individuals can be 'possessive' of patients – not wanting BHCs to be involved.<br>• *Confusion about/inconsistent warm hand-offs:* Inconsistencies from all professionals on how a warm hand-off is completed at their respective practice in their roles, including inconsistencies for communicating the BHC's role to patients.

No.	Study (year)/ Setting/ Country	Research Aim(s)	Condition(s)	Psychology Professional Group	Service/intervention(s) delivered	Research Methods	Participants	Key findings (relevant to views/experiences of patients and/or professionals on psychology provision in primary care)
							Centre manager (n=1)	• *A desire for more resources:* BHCs expressed a need for additional tools and/or resources to help them in their role (e.g. more educational opportunities (conferences, continuing education, case studies), greater access to psychiatric specialists, improved resources for patient engagement during treatment (for e.g. mindfulness, CBT). Nurse practitioners desired more BHC hours on site or increased BHC availability, and more educational materials on common mental health issues.<br>*Facilitators to behavioural health integration:*<br>• *Buy-in from physicians:* Physician support for integrated care improved BHC utilisation, saved time, and increased workflow efficiency. Some physicians incorporated BHC visits into warm hand-offs, highlighting recognition of BHC value. Rapport-building between BHCs and physicians was key to fostering buy-in and collaboration.<br>• *Increased physician capacity to treat common mental illnesses:* BHI initiatives, supported by education/training, enhanced primary care provider's ability to manage conditions. Collaboration with BHCs improved care provided to patients.<br>• *Colleagues as a key resource for staff:* Staff frequently relied on BHCs for guidance, training, and clinical support, often preferring these colleagues over external resources due to accessibility. Tools such as "cheat sheets" and instant messaging (IM) enhanced communication, enabled real-time consultation, and supported education on behavioural health care, pharmacotherapy, and crisis management.
12.	Brooks et al. (2016)<br><br>Federally qualified healthcare centres (FQHCs) (n=3)<br><br>USA	To examine patient and clinical utilisation and satisfaction of a brief treatment counselling toolkit in FQHCs.	Risky and problematic alcohol and illicit drug use	Behavioural health counsellor	• Either 1-session brief intervention (SBIRT) or expanded brief intervention, encompassing up to 5 additional in-person sessions (SBIRT+).<br>• Brief intervention toolkit assists counsellors to screen for risk use, and then conduct 35 separate interventions based on patient motivation and use severity.<br>• The toolkit includes communication strategies for providers to use with patients (e.g., educational cards and "talking points"), as well as brief training supplements to aid counsellors to implement the toolkit with fidelity.	Mixed-methods (qualitative interviews, quantitative outcome assessments)	**Qualitative**<br>Clinic staff (administrative, behavioural health and medical staff) (n=27)<br><br>Patients (n=6)<br><br>**Questionnaire**<br>Patients (n=274)	• Medical providers were generally favourable about working with counsellors providing SBIRT and brief treatment on site at the health centres.<br>• Behavioural health counsellors reported that providing standardised screening and brief treatment on-site changed their working relationships with medical providers around substance use: *"Clinically, it's changed the providers' mindset tremendously in terms of how much or how often they assess for substance abuse/misuse. They take it more seriously."*<br>• Patients found the meetings with the counsellors to be helpful, as many reported feeling isolated with no one to talk to about their problems. Patients voiced the perception that the clinical interventions were helpful and should be offered more widely: *"I wish these programs were at every doctor's office. I think it would be great because there are a lot of people out there like me."*<br>• Overall, patients were generally very positive that it was both helpful and comfortable to be asked about and further discuss their drug and alcohol use at the FQHCs.<br>• The majority of participants (95%) felt that the counsellor was 'Very Qualified', and 74% reported using the skills they learned in their sessions with the counsellor.<br>• Most participants (57%) reported receiving a referral for specialty services, but only 40.9% of those that received a referral attended any of those services.
13.	Budd et al. (2022)<br><br>General practices (across	To understand how trainee associate psychological practitioners (T/APPs) add value to	Patients who would benefit from mental health promotional or	Trainee Associate Psychological Practitioner (T/APP)	• 4x 45-minute appointments (one per week); 45-minute follow-up 4-6weeks later. Face-to-face, telephone, or virtual (patient preference).	Mixed-methods (clinical outcome measures, surveys with qualitative	General practice staff (e.g. general practitioners (GPs), practice	• 97% of general practice staff stated the addition of a T/APP practitioner had a positive impact on the service they worked in.<br>• 96% of T/APPs said they found that patients engaged with the support they offered.<br>• 78% of T/APPs said they would recommend the T/APP role to other psychology graduates.

No.	Study (year)/ Setting/ Country	Research Aim(s)	Condition(s)	Psychology Professional Group	Service/intervention(s) delivered	Research Methods	Participants	Key findings (relevant to views/experiences of patients and/or professionals on psychology provision in primary care)
	23 primary care networks (PCNs))<br><br>UK	mental health care in general practice settings.	preventative advice (delivered within the context of a brief intervention) e.g. stress, reduced wellbeing and/or common mental health symptoms (depression, anxiety).		• Psychological assessment in the first session, the second focuses on a structured psychological formulation.<br>• Psychological work and advice given during either the first and second session, but is the main focus for third and fourth session.<br>• Individual sessions informed by cognitive-behavioural theory, solution-focused theory, motivational interviewing, health coaching, compassionate mind principles, distress tolerance and mindfulness-based skills and systems theory.<br>• Within some areas, the T/APPs delivered the service across all practices within the PCN, elsewhere, T/APPs were located within one practice.	and quantitative sections)	managers, nurses) (n=33)<br><br>T/APPs (n=23)<br><br>Patients (n=240)	• Almost all patients were both accepting of and benefitted from the support they received from T/APPs. They liked the type of support (e.g. being person-centred). Patients highlighted the benefits of access (being in their local general practice, shorter waiting times than other mental health services).<br>• Patients suggested increasing the number of sessions to greater than four. Some patients acknowledged limitations in the support the T/APP was able to provide (as they are trainees) and suggested developing further skills may be of benefit.<br>• The T/APP role was received positively by practitioners (general practice staff and T/APPs) in terms of benefits to patients, the type of support offered, and accessibility. Practice staff highlighted that T/APPs presence enabled them to learn more about mental health/wellbeing.<br>• Staff suggested offering more than four sessions.<br>• Staff identified challenges with integrating T/APPs into primary care settings. Some T/APPs expressed feeling isolated and not fully integrated into the general practice team. T/APPs and PCNs acknowledged difficulties with practice staff understanding the function and remit of a T/APP, and highlighted practical issues such as finding room space for the T/APP. T/APPs and practice staff suggested increased communication may be helpful to resolve these issues.
14.	Caballol Angelats et al. (2023)<br><br>Primary care teams (n=11)<br><br>Spain	To explore the perceptions and experiences of health professionals who participated in a multicomponent program for fibromyalgia patients based on health education, physical exercise and CBT.	Fibromyalgia syndrome	Psychologist (along with a GP, physiotherapist, nurse)	• The intervention included 12-week/2-hour session group-based programme combining: health education, physical exercise, and CBT, based on pain and attention management, learning to manage emotions, strategies for coping with difficulty, and pleasurable activities planning.	Qualitative (interviews)	GPs (n=3)<br><br>Nurses (n=9)	• Professionals believed that the program was useful.<br>• The multidisciplinary approach was beneficial to patients.<br>• All the topics covered by the program were considered timely, necessary, and relevant. The number of sessions was considered to be adequate.<br>• The primary care centres were found to be an adequate space, however, one participant suggested that the most important consideration is for the place to be comfortable with little noise and a high degree of privacy. According to the professionals, the patients also had reservations about the lack of confidentiality in the primary care centres because people in their area usually know each other, especially in villages.<br>• Professionals believed that the accessibility of primary care would favour its future implementation.<br>• The professionals perceived that patients had benefited from participation in the program. They remarked that the patients became involved, learned valuable information and were satisfied with their experience of the intervention.
15.	Chomienne et al. (2011)<br><br>Family medicine practices (n=2, 1 rural, 1 urban)	To explore the perceived impact on doctors and patients, of having family doctors and psychologists work together.	Various (e.g. anxiety, depression, adjustment disorder, health-	Clinical psychologist	• One full-time salaried clinical psychologist was integrated into each practice for 1 year. Psychologists had >10 years clinical experience.	Mixed-methods (qualitative focus groups, quantitative questionnaire)	Patients (n=376)<br><br>Doctors (n=10)	*Patient findings:*<br>• 77% of patients found that the psychologist had more time and 75% considered the psychologist to be better trained than their family practitioner to handle their psychological problems.<br>• Patients felt that seeing the psychologist did not adversely affect their relationship with their doctor.

No.	Study (year)/ Setting/ Country	Research Aim(s)	Condition(s)	Psychology Professional Group	Service/intervention(s) delivered	Research Methods	Participants	Key findings (relevant to views/experiences of patients and/or professionals on psychology provision in primary care)
	Canada		related problems, stress)		• Patients could self-refer or be referred by their doctor.<br>• Psychologists conducted assessments, consultations and short-term treatments (8-12 one-hour sessions). Psychologists recorded clinical impressions, treatment plans and progress notes in the common charts.<br>• To initiate contact and foster collaboration, psychologists organised for doctors, daily scheduled 'open door' hours and offered unscheduled consultations.<br>• Study included 4x90-minute knowledge transfer sessions between psychologists and doctors.			*Doctor findings:*<br>• 7/9 doctors reported that collaboration with a psychologist gave patients access to care they needed.<br>• 9/10 felt that their knowledge of psychologists' work/methods improved, although the majority (7/8) continued to feel ill-prepared to deal with psychological problems.<br>• All doctors rapidly developed a good working relationship with the psychologist, and rated the quality of feedback from the psychologists (e.g. discussions, recommendations, notes in charts) as excellent.<br>• Doctors agreed that having access to a psychologist resulted in earlier diagnosis and management of mental health problems. The ability to refer patients for rapid assessment and intervention had a major positive impact on their practice.<br>• The doctors felt the quality of care improved and that the intervention freed up their time. Most doctors reported improved office atmosphere and quality of life at work (8/10) as well as improved workload (7/10).<br>• 9/10 doctors agreed that psychologists should continue to be integrated into family medicine clinics on a full-time basis and one on a part-time basis.
16.	Cordella et al. (2016)<br><br>Primary care practices (n=4)<br><br>Italy	To investigate whether the presence of a psychologist in the primary care setting together with the physician affects the image of psychologists among the public.	Various (patients recruited from the waiting room)	Psychologist	• The service does not cater exclusively for individuals with "mental distress" or "mental illness," but for the whole population, just as physical disease services do.<br>• The aim of the service is to solve problems (existential, psychological).<br>• In two of the practices physicians worked once a week in the presence of the psychologist, while in the other two the physicians worked alone.	Observational (questionnaire with open and closed questions)	Patients (n=214)	• 65% of all the participants believed that the psychologist addresses daily life problems and 93% considered the psychologist to be generally useful. However, only 28% of the participants considered the psychologist to be useful to themselves, while the remaining 72% of the sample considered the psychologist to be useful to people other than themselves.<br>• Patients who attended a joint consultation with the psychologist and physician were four times more likely to perceive a personal relevance than those who consulted the physician alone.
17.	Cos et al. (2022)<br><br>Federally qualified health centres (FQHCs) (n=40)	To examine the ability to sustain integrated primary care behavioural health (e.g., co-location, communication and coordination) in 40 community health centres, during the COVID-19 pandemic and	Not reported	Behavioural health consultant (BHC)	• During the pandemic, BHC visits were provided by telehealth between 50-100% as a percentage of visits.<br>• Overwhelmingly, telehealth was provided by telephone; however, video was offered by two-thirds of sites. Patient preference, followed by limited patient	Qualitative (interviews)	Behavioural health consultants (BHCs) (n=55) across 40 unique FQHC sites	• Electronic health record (EHR) messages and/or 'tasking' were commonly used between primary care providers, staff, and BHCs for referrals. When on site, in-person handoffs also used.<br>• Some sites allowed BHCs to cover multiple sites within their agency and used a 'proxy box' in the EHR, where referrals would be submitted and the next available BHC would take the referral, regardless of 'home' site. Most BHCs managed and conducted their own scheduling, but providers also could add patients to the BHC schedule directly.<br>• 'Cold' call visits or direct outreach to patients, in the exam room or waiting room, were reported to be logistically harder to manage post-COVID. In response, BHCs reported

No.	Study (year)/ Setting/ Country	Research Aim(s)	Condition(s)	Psychology Professional Group	Service/intervention(s) delivered	Research Methods	Participants	Key findings (relevant to views/experiences of patients and/or professionals on psychology provision in primary care)
	Independent FQHC partnering agencies (n=10)<br><br>USA	review adaptations and challenges to provide integrated behavioural health via telehealth.			access to streaming video, were the primary reasons cited for telephone.<br>• Sites varied greatly whether the BHC was exclusively located in the office (20%), split time between on-site and remote (30%), or was remotely based (50%).		Agency that manages at least one FQHC (n=10)	using the following strategies: stopping doing cold calls; using population health applications to 'hotspot' patients who could most benefit from outreach; using the daily provider EHR schedules as guides to whom they could outreach.<br>• BHCs noted it has been harder to connect patients to outpatient behavioural health and experienced disruptions in care for those connected to outpatient treatment, leading to more collaboration and creative problem solving between the BHCs and their primary care providers to meet patient needs.<br>• Interdisciplinary team meetings (e.g. complex case discussions) were becoming common across agencies prior to COVID-19. As a result of COVID-19, meetings were largely on hold. BHCs reported that sites were trying to restore huddles and interdisciplinary meetings in late 2020. All agencies reported that sites maintained their usual supervisory and BHC department meetings.<br>• BHCs felt remote work decreased the sense of community with their teams, creating a feeling of loss and not being able to have workplace traditions (e.g. lunch, mutual support). BHCs also noted the loss of huddles and opportunities to collaborate with the team in meetings and informal curbsides. At one agency where BHCs had remained on site, the BHCs reported increased comradery with their team, noting the shared experience working together through COVID-19 had brought them closer.<br>• BHCs felt that core aspects of integration were well maintained and primary care behavioural health integration remained strong, as highlighted by: increased referrals by primary care providers; improved BHC penetration rates into the FQHC panels; back-and-forth communication (e.g., 'closing the loop') about the BHC visit between the primary care provider and BHC; increased utilisation of chat applications to enhance collaboration and informal communication.<br>• In an annual integration assessment with different disciplines (e.g. medical/nursing, BHCs, operations), there was a decrease ($p>0.5$) during the pandemic in co-location, clinical delivery and overall score, largely based on BHCs being mostly off-site, the lack of integrated care planning meetings, and interruptions in clinic behavioural health screening. However, due to agency-led site planning for pandemic operations and implementation of remote BHC telehealth, as well as frequent referral-specific BHC primary care provider communication, FQHCs demonstrated heightened features of integrated practice organisation.<br>• BHCs reported increased demands of higher productivity, higher patient acuity and the societal impacts of COVID-19 closures and remote work and often felt more stressed and more limited in enacting their usual coping repertoire. With increased stress and reduced separation between work and home life during social isolation, BHCs reported the need to actively focus on addressing their own stress management and setting boundaries with work demands. BHCs at three agencies noted they had taken on significant additional roles and initiatives in addressing their team's self-care. This ranged from healthcare staff seeking out the BHC to ventilate stress and obtain behavioural health referrals for themselves, to BHCs conducting mindfulness activities before team-wide meetings, to

No.	Study (year)/ Setting/ Country	Research Aim(s)	Condition(s)	Psychology Professional Group	Service/intervention(s) delivered	Research Methods	Participants	Key findings (relevant to views/experiences of patients and/or professionals on psychology provision in primary care)
								another agency providing a daily BHC-led support group for staff that served as a forum for training on coping skills, providing education and resources and evoking laughter.<br>• Anecdotal reports noted a number of patients who previously had declined/no-showed for a BHC visit subsequently completed a telephone meeting with the BHC and cited the phone (instead of in-person) as a major reason why they completed the visit. No-shows were largely reported to be reduced across the agencies.<br>• BHCs held a favourable opinion of telehealth and saw an important role in its continuation. BHCs appreciated the ability to reach a wider range of individuals and it was favourable to patients. Patients appreciated convenience, it reduced care barriers (e.g. travel time/costs, wait times, needing to take time from responsibilities), and some patients felt seeing their medical provider and BHC in the same day on-site was overwhelming. BHCs reported that patients seemed better prepared for visits, were more comfortable than in the office, and establishing rapport was easier than expected.<br>• BHCs reported frustrating aspects of telehealth e.g. patients forgetting about the appointment when called (but still happy to go on with the visit); a high number of calls to reach some patients; some individuals did not have phone access and/or privacy to discuss personal topics; some patients were multitasking during sessions (which necessitated assertive confrontations by the BHC); and it could be difficult to do calls with young children/families. BHCs also reported learning: more about patients/their context through telehealth, they could conduct high-quality behavioural health interventions over the phone/video, and were able to develop strategies to help patients access handouts and resources that would normally be provided by hand in the office.
18.	Dath et al. (2014)<br><br>Primary care practice (n=1)<br><br>New Zealand	To evaluate the outcomes of the provision of evidence-informed psychological therapy in a primary care setting by a clinical psychologist (who mostly worked in secondary mental health and provided outreach services to primary care on a weekly sessional basis).	Mild-moderate mental health disorder	Clinical psychologist	• After initial assessment, patients undertook a time-limited (4 session) individually tailored psychological intervention targeting mood improvement based on CBT and associated skills-based techniques e.g. problem-solving, lifestyle enhancement, behavioural activation, emotional regulation techniques, interpersonal/ relationship effectiveness, or other interventions negotiated with the patient following discussion of the psychological formulation, needs, and therapeutic goals. The patient's GP was informed about the diagnosis, treatment plan, and	Mixed-methods (clinical outcome measures, patient satisfaction survey, primary care staff interviews)	Patients (n=not reported)<br><br>Primary care staff (8 GPs, 1 practice nurse) (n=9)	• The client satisfaction survey found that 93% (13 respondents) indicated being very or mostly satisfied with their psychology treatment and 100% (14 respondents) reported the treatment helped them manage their mental health problems better. 85% percent of patients reported being very or mostly satisfied with the number of sessions they received. Barriers to seeking help earlier were: concern about cost (43%), not aware of how to access services (36%), and stigma (21%). 100% reported that they would prefer to receive therapy at their GP clinic rather than go to a mental health service.<br>• Qualitative results indicate the program improved timely access to mental health care, assisted GPs to manage the mental health needs of their patients better, and increased how well patients' mental health needs were addressed. Eight of nine (89%) primary healthcare staff reported this project improved access to therapeutic services and added additional resource for managing patients' difficulties. One GP reported not referring any patients due to not identifying any suitable candidates.<br>• Staff perceived the project as supporting improved mental healthcare through: easy and inexpensive access to psychological services that patients otherwise could not have accessed; improved uptake because it was offered in the familiar and non-stigmatising environment of the GP practice; improved support for GPs and practice nurses to meet the healthcare needs of their mental health patients; the synergistic relationship of the psychologist being able to extend the work primary healthcare staff could do and the primary healthcare staff being able to reinforce the psychologist's work with the patient;

No.	Study (year)/ Setting/ Country	Research Aim(s)	Condition(s)	Psychology Professional Group	Service/intervention(s) delivered	Research Methods	Participants	Key findings (relevant to views/experiences of patients and/or professionals on psychology provision in primary care)
					recommendations for additional support by primary care staff.<br>• The 4-session intervention involved a 90-minute initial assessment, two one-hour sessions at two-weekly intervals, and a final one-hour session including completion of outstanding aspects of therapy, relapse prevention, and therapy termination.<br>• Self-help resources (e.g. free online mindfulness training; stress, anxiety or depression management packages) were introduced as appropriate.<br>• Depending on patient need, up to two telephone follow-ups were undertaken by nurses (providing support, encouraging use of therapeutic strategies, and enhanced motivation).			and the ability to catch mental health problems earlier when adequate treatment takes less time. The project was perceived as improving communication between primary care and secondary mental health services.<br>• The disadvantages or limitations identified were: the availability of sessions only during working hours, the restricted number of sessions was sometimes insufficient, and that patients on other specific chronic care management programmes could not access the service. One GP also speculated that a few people may find it stigmatising to receive this kind of care through their GP practice.
19.	De Master (2011)<br><br>Various psychologist practice settings including primary care<br><br>USA	To examine the effects of educational experiences and professional practice on collaborative practices with and attitudes toward primary care.	Not reported	Psychologist	Not reported.	Quantitative (survey)	Psychologists (n=104)	• The findings suggest openness among psychologists to collaborative care with primary care providers but a difficulty engaging in collaborative practices unless having past or current experience within a medical setting.<br>• Psychologists who had current or past professional practice in medical settings were found to endorse significantly more collaborative actions taken on management of client cases referred from primary care providers.
20.	Dueweke (2019)<br><br>Primary care clinics (n=4)<br><br>USA	To evaluate the preliminary outcomes, acceptability, and feasibility of a brief crisis response planning intervention for patients at a moderate risk for suicide in a primary care behavioural health setting.	Moderate risk of suicide	Behavioural health consultant (BHC)	• Behavioural health services integrated into practices full-time (n=3) and part-time (n=1).<br>• In-depth risk assessment with patient conducted.<br>• The patient worked with the BHC to collaboratively create a crisis response plan in their initial visit.<br>• Patients were given a copy of their individualised crisis	**Patients**<br>Quantitative (pre- and post-self-report measures)<br><br>Qualitative (interviews)<br><br>**BHCs**	Adult primary care patients at moderate risk for suicide (n=22), 16 participated in follow-up interviews<br><br>BHCs (n=3)	*Patients were asked what they found helpful about the visit:*<br>• 10 patients mentioned specific characteristics about the BHC (e.g. that the BHC was kind, calm, helpful, supportive, reasonable).<br>• Safety plan components were found to be helpful (e.g. coping strategies etc.) (n=8).<br>• The service was seen as a positive alternative to long-term therapy, hospitalisation, or medication (n=3).<br>• Patients felt empowered (n=3), had increased hope (n=3), and that the visit normalised talking about suicide (n=2). One patient mentioned that the most helpful part was when the provider helped connect her with resources (e.g. a referral for long-term therapy).

No.	Study (year)/ Setting/ Country	Research Aim(s)	Condition(s)	Psychology Professional Group	Service/intervention(s) delivered	Research Methods	Participants	Key findings (relevant to views/experiences of patients and/or professionals on psychology provision in primary care)
					response plan to take home at the end of the first visit.<br>• A copy was also scanned and uploaded to their electronic medical record.<br>• Upon completion of the initial visit, patients were scheduled for follow-up visits as necessary, and continued to receive treatment as long as clinically indicated.<br>• BHCs attempted to increase likelihood of follow-up by providing one reminder call prior to scheduled appointments, and calling patients to check-in and reschedule if they no-showed their follow-up appointment.<br>• Patients returning to subsequent behavioural health visits received interventions focused on re-assessing risk and use of the crisis response plan, as well as learning new skills related to emotion regulation, cognitive restructuring, and behavioural activation.	Qualitative (interviews)		• One patient commented on how receiving a warm handoff referral from her primary care provider helped ensure that she attend her behavioural health appointment.<br>• However, one participant disliked the brevity and limited frequency of visits within the behavioural health model, and another disliked the procedure of being referred to the suicide crisis team at a university counselling and psychological service clinic (n=1).<br>*BHCs were asked how the intervention fit with primary care:*<br>• All BHCs acknowledged that completing the risk assessment took longer than a typical 30-minute behavioural health visit. Despite taking longer, two BHCs felt it was feasible to offer this in the primary care setting and it was easily implemented.<br>• Two of the BHCs described adaptations they made to improve patient care, such as working with clinic staff to reschedule patients whose appointments had to be cancelled or delayed in order to accommodate the longer meetings with patients.<br>• Two of the BHCs stated that even though the safety planning intervention took extra time, the utility of the intervention superseded any scheduling concerns they might have.<br>• Two BHCs did mention specific difficulties of offering this intervention in primary care (e.g. one BHC said it was difficult being the only behavioural health provider in her clinic).<br>*BHCs were asked how did primary care providers respond to their management of suicidal patients with the intervention:*<br>• Primary care providers were positive. Two BHCs mentioned specific ways this intervention has benefitted primary care providers e.g. this has lifted the burden of risk detection and management from the primary care provider.<br>• Collaborative processes between BHCs and primary care providers emerged as a result of the intervention.<br>• Two BHCs mentioned primary care providers were often surprised to learn about the suicide risk BHCs had discovered through doing the risk assessment with their patients.
21.	Durcan (2020)<br><br>General practices (n=3)<br><br>UK	To describe promising approaches in local areas where clinical psychology has been provided in a primary care setting.	Various (e.g. trauma, depression or low mood, anxiety, chronic physical health problems, symptoms of chronic pain)	Clinical psychologist, with some trainee psychologist support	Catterick practice (North Yorkshire):<br>• 15-minute appointments (option of 30-minute slot)<br>• Face-to-face and telephone consultations<br>• Psychologist worked in the practice<br>Ludlow and Telford practices (Shropshire):<br>• One psychologist in each area two days per week in each practice<br>• 30-minute appointments<br>• Face-to-face and telephone consultations	Mixed-methods (qualitative interviews, local audit data)	Stakeholders (GPs, clinical psychologists, service managers, commissioners and patients)	• All three sites reported positive feedback from both patients and families and found professionals in primary care to be very enthusiastic about the services offered e.g. *"…the patients and their families like the service…I get really positive feedback…"* (GP, Shropshire)<br>• In terms of referrals in Shropshire, the clinical psychologist felt they had made an impact on other services by ensuring onward referrals were only made when necessary: *"…the vast majority of referrals that we make are accepted and 'stick' with the service we refer to…"* This was due to detailed work being done by the psychologist in ensuring that the case was appropriate for referral, but also due to the psychologist preparing the patient on what to expect from secondary care (or other) services, so that they more were accepting of any offer made to them: this was described as "psychological readiness".<br>*Catterick local audit data:*<br>• Less than 5% of appointments offered were with a trainee psychologist, the vast majority were with the senior clinical psychologist.<br>• Of patients seen, 63% were female, 23% were aged 17 or less, 74% were 18-65 and 3% were over 65.

No.	Study (year)/ Setting/ Country	Research Aim(s)	Condition(s)	Psychology Professional Group	Service/intervention(s) delivered	Research Methods	Participants	Key findings (relevant to views/experiences of patients and/or professionals on psychology provision in primary care)
					In both services, professionals or patients could freely and directly book appointments to see the clinical psychologist. In the Telford service, the majority of appointments were booked by the patient's GP and fewer appointments were booked for the under-18 age range. In Catterick and Ludlow, however, appointments were mainly booked by the patients and there were a greater number of young people accessing the service.			• Referrals to services in the local mental health trust reduced over the pilot by 27%, and to community mental health teams by 47%. Referrals also reduced to local Improving Access to Psychological Therapies service (IAPT) (60% reduction). However, some services received an increase in referrals (e.g. Child and Adolescent Mental Health Services (CAMHS) increased by a third, but all those referrals were accepted). Referrals to a separate primary care mental health service also increased by 29%.<br>• Virtually all onward referrals (mental health and non-mental health) were accepted. So, whether the Catterick service reduced referrals to some services or increased them to others, it appeared to significantly increase the likelihood of any onward referral being accepted.<br>• Referrals from the practice in Catterick to secondary care have increased since the closure of the project and it was estimated this increase would be 28% by the end of the year post project.
22.	Ellbin et al. (2024)<br><br>Primary health care centres (n=15)<br><br>Sweden	To explore psychologists´ involvement and experiences regarding the organisation of the care process and treatment of patients seeking care for stress-related exhaustion.	Stress-related exhaustion	Psychologist, psychotherapist	Services/interventions broadly include:<br>• Assessment<br>• Psychoeducation (to increase patient understanding of stress reactions and associated symptoms to help the patient to structure daily activities)<br>• Psychotherapeutic work<br>• Manual-based group treatment.	Qualitative (interviews)	Psychologist (n=11)<br><br>Psychotherapist (n=4)	*Patient care*<br>• Many believed that the decision regarding which care the patients are offered is dependent on the physician or psychologist they happen to meet.<br>*Referrals*<br>• Reason behind the referral from the physician not always clear. Lack of plan in primary health care centres for the care process of exhaustion patients. Since many psychologists have a waiting list, the participants believed that patients were instead referred either to other services to speed up the process for the patient.<br>• Some participants discussed how referral from other professions to psychologists is done to solve a problem as a quick fix, without considering the overall process for the patients. This could lead to the patient lacking motivation or the psychologist struggling to identify a suitable focus for the treatment.<br>*Communication*<br>• Communication was raised as a prerequisite for good collaboration between different healthcare professionals.<br>• Most primary health care centres do not have a structured system with regular meetings to discuss and coordinate the patients care process.<br>• At some primary health care centres, psychologists are situated in a specific part of the centre and thus work relatively isolated with poor insight into the work of others, both within the psychologist group and among other professionals. However, at some primary health care centres, psychosocial teams had regular meetings where referrals were discussed, and patients were assigned to different professionals and/or treatment. Physicians seldom attend these meetings.<br>• Communication regarding patient care usually took place by reading each other's medical records, sending internal messages, or knocking on each other's doors. For most participants this worked well.<br>• Lack of collaboration, teamwork and a mutual approach led to an ambiguity regarding the patient's process through the care system. Sitting down, discussing, and reconciling

No.	Study (year)/ Setting/ Country	Research Aim(s)	Condition(s)	Psychology Professional Group	Service/intervention(s) delivered	Research Methods	Participants	Key findings (relevant to views/experiences of patients and/or professionals on psychology provision in primary care)
								patient matters, having a mutual mindset, and a clear treatment plan, is believed to provide a better overview of the patient's treatment.
23.	English et al. (2024)<br><br>Primary care practices (n=not reported)<br><br>USA	To explore the relationship between rural integrated behavioural health (IBH) and integrated primary care (IPC) practice adoption, job satisfaction, and provider burnout.	Not reported	Behavioural health provider	• Not reported	Quantitative (survey)	Rural medical and behavioural health providers (n=147)	• *Higher integration IBH/IPC integrated linked to improved staff well-being:* higher reported levels of integrated behavioural health (IBH) or integrated primary care (IPC) practices were significantly associated with lower emotional exhaustion (reduced burnout) and higher job satisfaction for staff.<br>• *Focus on rural context:* The findings are particularly valuable to add to the evidence for rural and primary care settings; underscoring that integrated care models can promote provider satisfaction and reduce emotional burnout within rural areas.
24.	Enos (2016)<br><br>Primary care practices (n=12)<br><br>USA	To report findings of mental health integration in primary care practice sites.	Various (e.g. depression, alcohol use disorders)	Psychologist	• Mental health specialists (which include psychologists) are stationed in the primary care practices throughout business hours to meet with patients.<br>• Appointments either together with physician or in separate office.	Case report	Health system chair of psychiatry (n=1)	• Anecdotally, the mental health specialists' presence is leading toward seamless care delivery that is averting the need for emergency mental health care.<br>• There is a more intense pace of activity in the primary setting, with the work involving *"quick interventions, quick responsiveness and quick decision-making."*<br>• Good communication and effective handoffs between primary care and behavioural health are essential.<br>• *"Most of the patients like this model of integration."* Although, a bridge program needs to exist for patients who are not immediately comfortable with the idea of receiving primary care and behavioural health services under the same roof.<br>• *"Having a patient show up in primary care and have behavioral health checking in, this is less stigmatizing."*<br>• The presence of behavioural health specialists is giving physicians and patients a preferable alternative to outside referrals, for which a patient likely would not be able to see a provider for a couple of months.
25.	Falanga & Pillot (2020)<br><br>GP practice (n=1)<br><br>Italy	To ensure all patients had direct access to a psychologist during treatment, even if they had not put in a specific request for one, to take care of accidental crises in real time,<br>to reduce spending on inappropriate pharmaceutical prescriptions and diagnostic examinations, and to facilitate health promotion.	Various (all patients attending the GP on a Thursday)	Psychologist	• The psychologist sat next to the GP in their office every Thursday, for two consecutive years.<br>• Psychological listening for each person visiting the GP practice.<br>• Exploration of the significance of any request.<br>• Psychological frameworking of the observed situations.<br>• Exploratory intervention when needed.<br>• Eventual further meeting in the GP practice for individual interviews.<br>• Sending a proposal for an additional visit, approved by the	Mixed-methods (observational, experimental, quantitative)	Family doctor (n=1)<br><br>Psychologist (n=1)<br><br>Patients (1,300 consultations over two years)	• Psychologist presence acceptable to patients.<br>• In the space of two years, only one person asked to be seen by the GP alone.<br>• In certain situations, patients were given the opportunity to have an individual appointment with the psychologist, in order to deepen what was introduced and to better explore the psychosocial aspects and the relational or life experiences of the patients.<br>• During the appointments that included the co-presence of the psychologist and GP, there was a 6% decrease in the rate of expenditure for medical investigations (hematochemical examinations, specialist visits, instrumental investigations) compared to the previous period.<br>• The daily defined doses prescribed by the GP decreased by 7%, with a reduction by 10% in pharmaceutical expenditures, compared to the period prior to the presence of the psychologist.

No.	Study (year)/ Setting/ Country	Research Aim(s)	Condition(s)	Psychology Professional Group	Service/intervention(s) delivered	Research Methods	Participants	Key findings (relevant to views/experiences of patients and/or professionals on psychology provision in primary care)
					GP and the patient, if necessary, to the mental health specialist.<br>• Debriefing with the GP at the end of the day.			
26.	Farb et al. (2017)<br><br>Primary care settings (n=6)<br><br>USA	To analyse the implementation of behavioural health integrated in six New Orleans Charitable Health Fund organisations.	Not reported	Behavioural health staff	Behavioural health integration based on recognised models:<br>• Camden Coalition: A care coordination model that uses data to identify a small subset of high-needs, high-cost patients, and dedicates resources to address the problems causing high-cost, poor-quality outcomes.<br>• IMPACT: A care delivery model that involves team collaboration with a shared care plan, population-based care management, evidence-based treatments, a treat-to-target approach, and shared accountability for patient outcomes and care processes.<br>• PCBH: A care delivery model that positions a behavioural health provider in the primary care exam room to function as a member of the primary care team and act as a liaison between the primary care team and behavioural health care team.	Qualitative (interviews)	Provider and staff (e.g. administration, behavioural health staff, primary care staff) (n=27)	• *Systems integration:* Challenges with implementing systems changes, including insufficient time and competing workflows, inconsistency in screening frequency and use of tools, and resistance from primary care staff.<br>• *Beliefs and commitment:* Although staff investment and commitment to behavioural health integration was improving, staff buy-in did not always translate to action, highlighting the importance of implementation infrastructure and processes in addition to increased buy-in. Changing organisational culture to increase staff commitment was commonly reported as a challenge.<br>• *Integrated practice:* Collaboration between primary care staff and behavioural health staff improved as the organisations further involved behavioural health staff in care teams and primary care provider meetings. Collaboration increased via evidence-based formalised channels (e.g. warm hand-offs) and informal channels.<br>• *Shared decision-making:* Organisational culture change led to behavioural health staff increasingly being seen as providers on the care team. Several behavioural health staff respondents reported frustration that they were still seen as auxiliary to primary care staff. Respondents at one organisation noted that behavioural health staff had to prove themselves, with one self-advocating to have a more involved role. A respondent from another organisation speculated that resistance to sharing authority came from primary care providers having limited previous exposure to behavioural health staff.<br>• *Relationships:* Increased collaboration resulted in primary care staff and behavioural health staff feeling more comfortable working together with increasing respect and trust.<br>• *Training:* Respondents expressed a need for increased training (e.g. in integrated care, and behavioural health staff and primary care providers providing training for each other).<br>• *Leadership:* Findings highlighted the importance of organisational leadership, as several organisations attributed an increase in staff commitment to leadership support. A behavioural health integration champion also facilitated the implementation process. Leadership and administration, clinic processes and systems, and provider relationships were critical components of successful behavioural health integration implementation.

No.	Study (year)/ Setting/ Country	Research Aim(s)	Condition(s)	Psychology Professional Group	Service/intervention(s) delivered	Research Methods	Participants	Key findings (relevant to views/experiences of patients and/or professionals on psychology provision in primary care)
27.	Farmanova et al. (2017)<br><br>Family medicine practices (n=2)<br><br>Canada	To examine the development of collaboration between primary care physicians and psychologists integrated in two community-based primary care practices for a one-year project.	Not reported	Psychologist	• Physicians could refer patients to the psychologist, filling out a referral note or the patient could self-refer.<br>• The psychologists were requested to keep open hours once a week to encourage physicians to stop by and discuss care plans and other aspects of patient care and collaboration.<br>• The physicians were requested to participate in four 90-minute knowledge transfer sessions (accredited by the Canadian College of Family Physicians) delivered by psychologists every three months.	Qualitative (focus groups/ interviews)	Psychologists (n=2)<br><br>Physicians (n=14)	*Inception phase (month 0 to month 6):*<br>• Both physicians and psychologists reported that collaboration remained unstructured. Physicians were concerned of the short study duration, likely attributed to the study as an opportunity to increase access to psychological care for patients who could not afford it. Some physicians described psychologists as ‘caregivers’ not only to patients but also to themselves, and were concerned their departure would have a negative effect on their practice (e.g. increased stress from having to handle mental health conditions).<br>• Psychologists described the pace of work as quicker than they were used to, and acknowledged how busy physicians were. They reported having a higher caseload than in a private practice. They were concerned that physicians might be taking on cases that could be referred to them. They also perceived they were intruding on physicians' time when they desired to discuss cases with them. However, this dissipated by the end of the inception phase.<br>• Psychologist open hours were removed by the end this phase as physicians did not take advantage of them, preferring brief and casual hallway consultations.<br>*Maturation phase (month 7 to month 13):*<br>• More mature collaboration was established between physicians and psychologists.<br>• The classic form of referral and consultation (referring and receiving feedback in the form of a written report) was described as the standard form that had taken place most frequently. Referrals tended to be either for treatment alone or for diagnosis and treatment. Informal collaboration/corridor consultation was described as complimentary to the classic form of referral and was commonly used to inform colleagues of progress and general case impressions; to explain/clarify diagnoses or an entry/comment in a patient's file; to consult regarding a condition; to find out if a referred patient saw the psychologist or terminated treatment early; and to provide/obtain updates on complex cases (e.g. suicidal tendencies).<br>• Physicians and psychologists reported an increase in two-way communication. Communication was supported via messaging and emails within the electronic medical record (EMR) and this use increased as the project advanced. Messaging/emailing within the EMR kept communication open because as it ensured the message was received.<br>• Classic referral and informal consultation were viewed as most realistic/practical forms of collaboration in the primary care context. Collaboration did not advance beyond classic referral and informal consultation during the project. This was attributed to lack of time, use of routine practices such as referrals, and privacy issues.<br>• Knowledge transfer sessions delivered by the psychologists were viewed as a substitute for formal collaboration.<br>• The EMR to communicate and exchange information was seen as an effective collaboration vehicle. Access to the psychologist's notes reduced the need to meet in person to discuss patient care. Electronic notes were regarded as better than an informal hallway consultation as 1) it ensured privacy 2) notes facilitated reflection as they tend to be more detailed than a brief hallway exchange.

No.	Study (year)/ Setting/ Country	Research Aim(s)	Condition(s)	Psychology Professional Group	Service/intervention(s) delivered	Research Methods	Participants	Key findings (relevant to views/experiences of patients and/or professionals on psychology provision in primary care)
								• Physicians and psychologists identified similar qualities as important for a good collaborative process:<br>○ Communication and access to each other.<br>○ Being comfortable with each other (described as when working in the same location).<br>○ Confidence in each other's professional competence was perceived as a prerequisite for an effective referral system.<br>○ Mutual respect (recognition of the legitimacy and importance of the other's profession, seeing each other as equals in healthcare).<br>*Return to "care as usual" (month 14 to month 17):*<br>• Physicians stated that re-adjustment to usual care was difficult because they *"have lost a vital resource*".<br>• The main contribution of psychologists as perceived by physicians, was providing patients with care from the professional best qualified to treat them.<br>• Physicians identified several impacts resulting from the collaboration: improvement in referral process; improved access/affordability of care to patients; reduction in physician workload; reduction in personal stress level; increased satisfaction/pride that the clinic could provide adequate care to patients.<br>• Physicians stated that the collaboration experience left lasting effects on patients and the practice, despite returning to a pre-collaborative environment. Patients who had received treatments continued to improve. Physicians felt more confident to identify, deal, and refer certain psychological problems (likely related to the four 90min knowledge transfer sessions, topics of which psychologists and physicians determined together).<br>• The project had an impact on physicians' referral practices. Some stated they were now more likely to request feedback or a report in order to be kept in the loop, from the psychologist to whom they referred a patient in the community.<br>• Physicians expected reluctance from patients to be referred to a psychologist, but this was not the case. As a result, they referred more patients than they expected to do so at the start of the project.<br>• Physicians and psychologists indicated they had a sense of shared responsibility for the patients.<br>*Recommendations to improve collaborative process (as described by the authors in the results):*<br>• Any procedures or activities (e.g. scheduled meetings, co-provision of care) related to more formal types of collaborations should be set out clearly before collaborative process begins.<br>• The time a psychologist spends in a clinic should be flexible and based on clinic needs. Evening hours a few days a week allows patients who may decline psychological treatments to attend.<br>• Physicians making referrals should follow-up with patients who stop sessions with the psychologist to find out the reason. A joint session with the referring physician, psychologist, and patient could help discuss the issue in a non-intimidating way. This would reinforce a team-based approach to care.

No.	Study (year)/ Setting/ Country	Research Aim(s)	Condition(s)	Psychology Professional Group	Service/intervention(s) delivered	Research Methods	Participants	Key findings (relevant to views/experiences of patients and/or professionals on psychology provision in primary care)
28.	Fletcher (2021)<br><br>Primary care practice (n=1)<br><br>New Zealand	To investigate if the impact of a co-located psychological service as a resource for primary care providers to refer patients with mild to moderate mental health needs would impact on the wellbeing of the providers at work.	Patient exhibiting mild to moderate mental health needs	Psychologist	• Focused acceptance and commitment therapy (FACT).<br>• 1-6 sessions, 20-30 minutes each.<br>• Referrals to the co-located FACT service could be made by GPs and nurses.<br>• Wherever possible the initial FACT appointment is on the same day as the appointment with the health provider. If the psychologist is unavailable or the patient seeks to delay, then a booking can be made through the facility's shared booking system.<br>• FACT services are provided on-site by six registered psychologists and is operational three full days and two half days per week.	Qualitative (interviews)	General practitioners (GPs), nurse practitioners and registered nurses (n=9)	• Participants indicated that the ability to refer patients to a timely, appropriate psychological service was beneficial for the patient.<br>• There was overwhelming support by participants for the FACT service, including the ability to get prompt help for patients, and the service was viewed as a positive experience for both the patient and the practitioner.<br>• Participants had different understandings of the referral criteria required for the FACT service.<br>• There was a lack of understanding about the deliverables of the FACT service with many commenting that the sessions were "rushed"; "too short"; or "not in-depth enough".<br>• Participants indicated that GPs should be delivering FACT services as part of a medical consultation.<br>• Participants felt that regular feedback from the FACT service's psychologists regarding the number of correct referrals into the service, and learning about the patient outcomes would have a beneficial effect on practitioner wellbeing. Participants also desired a stronger relationship with the psychologists who provided the FACT services.<br>• Most participants were supportive of the FACT service and were reluctant to criticise the process of referral and service provision.<br>• Participants spoke of expanding the service to different population groups who may be missing out (e.g. youth and the elderly).<br>• Practitioners noted that the ability to ensure patients were seen for psychological distress takes the worry away from themselves but also noted that patients might not be ready for immediate support.<br>• GPs noted that attendance at a FACT session prior to a medical consultation would benefit both the patient and GP in terms of focus.<br>• When discussing patients' expectations, one participant commented there were "one or two who didn't like it".<br>• Participants indicated that they were able to review patient notes and see whether the patient was attending sessions or that they were able to maintain an ongoing clinical/patient relationship.
29.	Gidding et al. (2014)<br><br>Health centres (n=10)<br><br>Netherlands	To compare barriers and facilitators experienced by GPs, practice nurses for psychosocial care, primary care psychologists and patients working in stepped collaborative care for depression as a contribution to person-centred healthcare.	Depression	Clinical psychologist	• Stepped collaborative care programme where an initial depressive symptom assessment conducted by the GP.<br>• Patients directed towards specific therapy options: minimal interventions (steps 1 and 2) for non-severe and steps 3 and 4 for severe depression and symptom severity monitoring with the Beck Depression Inventory-2 every month.	Qualitative (focus groups)	GPs (n=16)<br><br>Practice nurses (PNs) (n=4)<br><br>Psychologists (n=6)<br><br>Patients (n=23)	• All participants agreed that psychosocial disorders are complex and difficult to diagnose and manage in primary care.<br>• The patients said that they often wait a long time before seeking help and once they do seek help, the GP often underestimates their symptoms.<br>• The PNs and patients thought that complex cases should be diagnosed and treated by another professional when the GP feels uncertain.<br>• The psychologists considered GPs' diagnostics regularly as a barrier and questioned the GPs' ability to recognise and manage depression. The psychologists therefore usually ignored the GPs' diagnosis and restarted the diagnostic process. It then takes them valuable time to explain to the patient why they prefer a different diagnosis.<br>• The psychologists felt that, since GPs sometimes wait too long before referring and because of long waiting lists, symptoms worsen unnecessarily before people receive appropriate care. According to the GPs, the long waiting lists are partially caused by a

No.	Study (year)/ Setting/ Country	Research Aim(s)	Condition(s)	Psychology Professional Group	Service/intervention(s) delivered	Research Methods	Participants	Key findings (relevant to views/experiences of patients and/or professionals on psychology provision in primary care)
					• Steps 1 and 2: psychologist delivered components include e.g. mindfulness, psychoeducation, problem solving, brief psychotherapy, group courses.<br>• Steps 3 and 4: psychologist delivered components include e.g. psychotherapy.			shortage of psychologists, whereas the psychologists think the waiting lists arise from GPs referring patients who do not actually need psychological help, and believe that more psychologists would only lead to even more patients being referred unnecessarily.<br>• All participants identified the increased collaboration in the stepped collaborative care program and regular communication between different care providers as facilitators for high-quality care.<br>• The professionals suggest more communication via the digital patient records.
30.	Gomez (2017)<br><br>Primary care clinics (n=3)<br><br>USA	To gather qualitative self-report information from patients in order to obtain information regarding what they recalled about the services they received.	Various (mood, anxiety, sleep, grief, diabetes management, alcohol, stress, relationship problems, interpersonal violence)	Pre-doctoral clinical psychology graduate student (working as behavioural health intern)	• Behavioural health interventions were delivered during brief sessions lasting between 15 and 30 minutes.	Qualitative (survey)	Patients (n=83)	• Patients were asked to provide feedback about the behavioural health services they received. The most common response was that patients thought services received were good or great (44.6%). About a third of patients (33.7%) did not provide any feedback.<br>• There were some patients (12%) that reported services needed to be improved or reported they were not satisfied with the services they received.<br>• Some participants (7.2%) specifically noted that the providers had good qualities such as being kind, caring, nice, etc.<br>• Some patients (4.8%) gave specific feedback about the need to maintain the same behavioural health providers at each session or to have more frequent visits.
31.	Guarino (2020)<br><br>Family medicine practice (n=1)<br><br>USA	To test the feasibility and acceptability of a modified Dialectical Behaviour Therapy (DBT) skills-only group intervention in an integrated behavioural health setting.	Mental health concerns	Advanced graduate student in Clinical Psychology	• DBT skills group, 90-minute group sessions (either morning or evening).<br>• Rolling admission, five consecutive 10-week cycles, lasting for approximately 1 year. Patients encouraged to attend all sessions within a 10-week cycle.<br>• DBT skills including: mindfulness, emotion regulation, mindfulness to control attention, distress tolerance, interpersonal effectiveness.	Mixed-methods (questionnaires with quantitative and qualitative items and outcome measures)	Patients (n=35)	• Mean number of sessions attended per participant was 6.17 sessions (SD = 6.82). Reasons for termination included that participants were too busy to continue attending the group (20% of responses), with the second most common response being that participants had obligations that conflicted with the scheduled group times (18% of responses).<br>• Participants found the group to be overall enjoyable (mean = 1.88; SD = .33), helpful (mean = 1.88; SD = .33) and satisfactory (mean = 1.84, SD = .37).<br>• Results indicated a clinically significant reduction in PHQ-15 ($p<0.01$; d=.51), PHQ-9 ($p<.01$; d=.49), RSQ-RRS total ($p<.01$; d=.54), DERS total ($p<.05$; d=.40) and DERS clarity subscale ($p<.05$; d=.44) scores. Additionally, there was a clinically significant increase in DBT-WCCL skills use subscale scores (p=.001; d=.63).
32.	Hannah et al. (2012)<br><br>GP practice (n=1)<br><br>UK	To introduce the Nuka model of healthcare (integrated primary and community services) to a general practice in Scotland.	Not reported	Behavioural health consultant (health psychologist)	• A dedicated "Nuka" team within the practice to work using an integrated model.<br>• Nuka team: GP, practice nurse, nurse practitioner, administrator and behavioural health consultant (health psychologist) working in the same room for a panel of patients.	Mixed-methods (qualitative patient feedback in a meeting, staff satisfaction questionnaire)	Patients (n=not reported)<br><br>Staff (n=not reported)	• The Nuka GP undertook less than half of the number of face-to-face consultations compared to other GPs in the practice and performed roughly twice as many telephone consultations. The shift in activity represents more flexibility for the GP to spend longer with patients who need more time.<br>• Nurses in the Nuka team undertake many more consultations by phone compared to other nurses working in the practice.<br>• Staff wellbeing in the Nuka team has improved significantly from baseline on measures including decision making, team working, handling conflict, internal communication, work life balance, change and innovation.

No.	Study (year)/ Setting/ Country	Research Aim(s)	Condition(s)	Psychology Professional Group	Service/intervention(s) delivered	Research Methods	Participants	Key findings (relevant to views/experiences of patients and/or professionals on psychology provision in primary care)
								• Patients report they are pleased with the change in the service. *"Having used this service four times since the pilot began I have found this to be a much better and patient-friendly service. I can now get an on-the-day appointment without having the problem of trying to get through on the phone only to find out that all the appointments have gone. Even when it came to re-ordering my prescription this was ready on the same day of placing my order"* (Nuka Patient).<br>• Early results suggest the Nuka model of care changes the quality of interactions between staff and between staff and patients, improving access, delivering a better service and increasing staff satisfaction.
33.	Hard et al. (2015)<br><br>GP surgeries (n=not reported)<br><br>UK	To explore the context within which the service (City and Hackney Primary Care Psychotherapy Consultation Service (PCPCS)) functions.	Complex conditions characterised by co-morbidity and risk (e.g. medically unexplained symptoms, features of personality disorder, frequent attenders at GP surgeries)	Psychologist	• Offers a referral service for patients who require psychological provision.<br>• Includes a range of assessment options (usually 1-2 assessment meetings followed by a plan of action) and various treatment options of a brief, focused nature (up to a maximum of 16 treatment sessions).<br>• Treatment is based on a multi-model approach by utilising therapies informed by inter alia brief dynamic therapy, dynamic interpersonal therapy, mentalisation-based and cognitive behavioural models. Where appropriate, couple and family interventions are offered.<br>• Brief packages of group therapy, with a focus, e.g. health related concerns, are also offered.	Mixed-methods (outcome measures, questionnaires with qualitative items)	Patients (n=not reported)	• The PCPCS sends all patients a patient experience questionnaire a month following discharge from the service which asks *'Do you feel that the service has helped you to better understand and address your difficulties?'* To date, patients have answered the questions positively 80% of the time.<br>• Patients provided general comments about the service, which were mainly positive. The PCPCS responds constructively to comments and suggestions for improvement e.g. the service responded to concerns about it not being sufficiently flexible in the appointment times by offering some 'out of hours' appointments to individuals in employment, but the ability to do this is limited by surgery opening hours.
34.	Hartley et al. (2022)<br><br>General practice (n=1)<br><br>UK	Report describing clinical psychologists as GPs for mental health.	Various (e.g. anxiety, low mood, other mental health conditions)	Clinical psychologist (as a 'General Practitioner Clinical Psychologist' (GPCP))	• Clinical psychologist delivering a 'mental health GP' service, replacing GP sessions with GPCP sessions (provision 'instead of', not 'as well as' GP time).<br>• The GPCP sees patients first (not seeing patients who the GP has assessed and referred). The practice operated a triage system, where every	Mixed-methods (questionnaire, qualitative interviews, reflection)	Patient questionnaire (n=31)<br><br>Patient interviews (n=7)<br><br>GP interviews (n=5)	*Patient experience*<br>• *Satisfaction.* Four questions measured satisfaction (were expectations met; was contact with the GPCP helpful; overall experience; how likely to recommend the service). Patients were satisfied (all measures median scores of 4 out of a possible 5). Patient interviews revealed a key contributor to satisfaction was that they had more time to talk with the GPCP than in typical GP appointments.<br>• *Relevant referrals.* 11/31 patients were signposted by the GPCP to other services. Four patients rated the signposting 'extremely relevant', five 'somewhat relevant' and only two patients felt their referral was 'neither relevant or irrelevant' or 'irrelevant'.

No.	Study (year)/ Setting/ Country	Research Aim(s)	Condition(s)	Psychology Professional Group	Service/intervention(s) delivered	Research Methods	Participants	Key findings (relevant to views/experiences of patients and/or professionals on psychology provision in primary care)
					appointment request was rapidly assessed by a senior GP, who decides which clinician is best placed to see the patient at what level of urgency. The reception team then contact the patient to arrange the appointment. Patients were also able to request a GPCP appointment specifically.<br>• GPCP delivered timely, brief psychological interventions (e.g. motivational interviewing, solution-focused therapy, acceptance and commitment therapy (ACT) and focused ACT).		Reflections from the GPCP (n=1)	• *Confidence.* Patients discussed how a positive GPCP meeting enabled them to *'feel more confident about going to the GP with mental health issues'.* Due to increased confidence, some felt they no longer needed to contact other services for support.<br>• *Reassurance.* Patients felt reassured knowing that the GPCP was available.<br>• *Accessibility.* Patients valued how quickly they were able to access specialist mental health support. The GPCP location (based in patients' practice, within proximity to their homes) was beneficial in terms of reducing travel barriers.<br>• *Dissatisfaction.* As research was during COVID-19 pandemic, the majority of GPCP appointments were by telephone. Two patients, whilst satisfied with the GPCP, felt their experience could have been improved with a face-to-face appointment. The GPCP service was designed to be available 'as and when needed'. However, some patients advised they would have liked to have been followed up by the GPCP.<br>*GP experience*<br>• *Reducing workload.* Having the GPCP available was reported to have made '*a massive difference to [their] workload.*' GPs highlighted that their short consultation time is inadequate to understand patients', often complex, mental health issues, which limited the help they could offer to either simplistic solutions or medication. GPs reported over-running these appointments, creating time-pressure on their day. GPs felt the work of the GPCP affords GPs '*more time*' and appreciated being able to '*refer on to someone who's going to give the person more time... with their difficulty*'.<br>• *Emotional burden.* GPs spoke of the emotional pressure of responding to mental health need, and the negative impact on themselves and their work. Being able to share responsibility with the GPCP (with confidence the GPCP would be offering good care) reduced feelings of burden.<br>• *Accessibility.* GPs reported the GPCP service has served to '*enable people to access more timely psychological interventions*' in line with their needs, and GPs felt '*more positive that there is something to offer other than just tablets or referral into a void*.'<br>• *Onward referrals/other services.* GPs expressed frustration with rejected referrals by mental health services due to narrow eligibility criteria. The GPCP was able to see patients of any demographic, with a range of issues, including complex cases.<br>• *Additional* skillset. The practice gained from the GPCP's knowledge/training, with patients benefiting from a more psychological approach to consultations, and also as a useful source of advice for the GPs '*…having that skill set in the team is a huge asset, both to patients but, also, to us as we're talking about patients as well. So, I think our mental health offer... is much better and much more consistent*.'<br>• *Satisfaction with service.* All GPs were highly satisfied and believed that patients '*really valued [the service]',* expressing they would like to see the service expanded in the practice, in terms of both hours and scope. *'It's been valuable and I wouldn't want to go back without it'.* They were also positive about the other practices setting up a similar

No.	Study (year)/ Setting/ Country	Research Aim(s)	Condition(s)	Psychology Professional Group	Service/intervention(s) delivered	Research Methods	Participants	Key findings (relevant to views/experiences of patients and/or professionals on psychology provision in primary care)
								service, being *'something that a lot of practices would really benefit from'*, considering tailoring to the community and evolving in response to needs.<br>*Reflections from the GPCP*<br>• The role was about relationship building with patients, practice colleagues, and local organisations and charities. Also, finding ways of offering additional impact through assessment, formulation, intervention and consultancy skills.<br>• Being based at the practice helps to increase transparency of what psychologists do, both for patients and GPs.<br>• Initially, up to 15 patients were booked into the clinic each day for 20-30 minute consultations (many via telephone due to COVID-19). Clinical contacts were reduced to a maximum of 12 a day to slow down, and to improve assessment skills.<br>• The relationship with the patients is different to work in services offering therapy, as not starting therapy, and not working towards an ending.<br>• With patients, it's about establishing what has motivated them to get in contact and, as quickly as possible, get to an understanding of what they would like to be different about their life right now if their psychological difficulties were not getting in the way, and helping people open up to different perspectives and try small steps towards change in areas that are more in their control.<br>• Intervention approaches include: motivational interviewing, solution-focused therapy, the therapeutic model most aligned to is acceptance and commitment therapy, and focused ACT informed the approach to brief consultations.<br>• There are as few barriers as possible for patients to access the GPCP for further consultations. Focus on treating every session as a single opportunity to make a difference, although there is the paradox of the sessions being one-offs and yet unlimited (30% of patients returned across 20 months of GPCP service, of those 60% only returned once). The service will need to evolve and make it easier for patients to follow-up with the GPCP where needed.<br>• Mental health is impacted by the welfare system and work, housing, education, substance dependency, and trauma. Liaising with the practice's link workers are essential to the functioning of the GPCP role. The GPCP also writes letters and support patients with their contact with other organisations and services. There is work to do to develop better working relationships with the community groups, jobcentres, social workers and welfare rights advisors.

No.	Study (year)/ Setting/ Country	Research Aim(s)	Condition(s)	Psychology Professional Group	Service/intervention(s) delivered	Research Methods	Participants	Key findings (relevant to views/experiences of patients and/or professionals on psychology provision in primary care)
35.	Hepworth et al. (2015)<br><br>General practice (n=1)<br><br>Australia	To examine the impact of integrating mental health care service delivery into an urban Aboriginal and Torres Strait Islander primary health care service.	Prevention, management, and treatment of chronic disease (which can impact mental health and social and emotional wellbeing)	Psychologist	• The psychologist was physically located in a different building due to lack of available space in the clinic. Although this location contributed to privacy.	Mixed-methods (quantitative descriptive statistics, qualitative interviews and focus groups)	Interviews: patients (n=7), social work clients (n=5), practice dietician (n=1), social worker (n=1), psychologist (n=1)<br><br>Focus groups: GPs (n=10), PNs (n=6), Aboriginal health workers (AHWs) (n=5), receptionists (n=2)	• GPs referred 226 clients to the psychologist, and 199 mental health care plans were commenced.<br>• All participants reported improved access. Prior to this, clients had to travel approximately 16km to access services.<br>• Staff described inclusion of mental health services as enabling the provision of "holistic" and "culturally appropriate" care and having a positive impact on service users.<br>• Service users described their experience of accessing mental health care services in terms of the importance of supportive relationships and feeling "comfortable" in the environment.<br>• Health service staff talked enthusiastically about the strong relationships that underpinned the care provided by the practice, and attributed increased access to mental health services to several relational features.<br>• Patients were already familiar with the longstanding primary health care service and they had existing positive and trusting relationships with the health service staff that enabled greater comfort and ease of access.
36.	Hermens et al. (2014)<br><br>General practices (n=not reported)<br><br>Netherlands	To examine the gap between routine primary depression care and optimal care, as formulated in the depression guidelines, and to explore the facilitators and barriers that affect the provision of optimal care.	Depression	Psychologist	• Psychoeducation and counselling to mildly depressed patients.<br>• When treatment effects not satisfactory or patients more severely depressed, GPs offered, or referred to, psychotherapy or pharmacotherapy.<br>• Patients with complex and severe depressive disorder directly referred to specialised mental health care.	Mixed-methods (qualitative interviews/focus groups, quantitative questionnaire)	GPs (n=6) (GPs working in integrated practices (n=4))<br><br>Mental health care providers (n=22) (including n=7 psychologists)	• Considerable between-practice variation was found in the collaboration within primary care; some GPs closely collaborated with mental health nurses or with primary care psychologists, while other GPs only made referrals but did not collaborate, or rarely consulted other primary mental health providers.<br>• Close collaboration with mental health nurses or primary care psychologists seemed favourable for delivering optimal care.<br>• Most GPs indicated that the quality of care for depressed patients could be improved mostly by the strengthening of primary mental health care.
37.	Holt et al. (2022)<br><br>Nurse-Led primary care clinic (n=1)<br><br>USA	To identify factors which sustain integration of behavioural health in nurse-led primary care.	Behavioural healthcare	Behavioural Health Provider (BHP)	• 'Onboarding': Patients provided with description of service at a routine appointment.<br>• Health screening: Patients completed behavioural health screening (e.g. AUDIT; PHQ-9).<br>• Warm handoff: Post-primary care visit, nurses introduced patients to the BHP.<br>• BHP had a caseload and contributed to 'huddles', coordinated care plans etc.	Mixed-methods (qualitative feedback, quantitative surveys)	BHPs, psychiatrists, registered nurses, patients (n=not reported)	• The reported levels of integration increased over two years from 2.86 to 4.00 (on a scale of 1-6).<br>• Qualitative results found that the team embraced integration of behavioural health. Teamwork was found to be a critical component, and there are opportunities to utilise the skill set of team members to develop care plans. Further, the team were able to 'destigmatise' mental health concerns. Finally, patients praised the new model of care, with some expressing that all their needs were being met at one location.

No.	Study (year)/ Setting/ Country	Research Aim(s)	Condition(s)	Psychology Professional Group	Service/intervention(s) delivered	Research Methods	Participants	Key findings (relevant to views/experiences of patients and/or professionals on psychology provision in primary care)
38.	Howard (2012)<br><br>Primary care settings (n=not reported)<br><br>UK	To discuss the challenges faced by therapists working in primary care settings, relayed through personal experiences of the author.	Mental health	Clinical and counselling psychologists, psychotherapist, counsellors, IAPT trained therapists	• Psychological therapy (e.g. CBT).<br>• Collaborative care discussions.	Qualitative (personal reflection)	Author, psychotherapist (n=1) and generalised perceived experiences of therapists	• Team factors are seen as vital aspects of the experience of integration – including interpersonal dynamics and clear role responsibilities.<br>• Supervision is vital for therapists in primary care.<br>• Importance of structural support for psychology groups and the perception that they are vulnerable to exclusion by other team members.<br>• Seeing the psychologist as a core team member as opposed to a peripheral member of the primary care practice needs to be practiced.
39.	Hunter et al. (2018)<br><br>Primary care (n=not reported)<br><br>Not reported	Review Primary Care Behavioral Health (PCBH) model research on patient and implementation outcomes	Various	Behavioural health consultant	• All articles had to meet at least three criteria: (1) behavioural health services were delivered in primary care, (2) behavioural health services were available to all patients, and (3) patients were typically seen in appointments for 30 minutes or less.	Qualitative (literature review of PCBH model research including 32 manuscripts)	Patients and/or their caregivers (n=not reported)<br><br>Professionals (various) e.g. behavioural health consultants, nurses (n=not reported)	• Patients reported high levels of satisfaction with the PCBH model services, and they would seek this type of care again in the future, and would recommend it to others.<br>• There is high satisfaction of acceptability from the provider's perspective.
40.	Jewiss et al. (2023)<br><br>Primary care practices (n=8)<br><br>USA	To better understand contextual factors that support or impede behavioural health integration in primary care practices.	Chronic medical and behavioural health conditions (e.g. depression, anxiety, stress, insomnia, overeating, inactivity, smoking, medication non-adherence, chronic pain, problem drinking, substance use disorder, family distress)	Behavioural health provider	• Delivery of behavioural health integration: a collaborative system of care wherein behavioural health providers work closely with medical providers in patient assessment and management.	Qualitative (interviews)	Practice leaders (n=14)<br><br>Behavioural health providers (n=13)<br><br>Medical providers (n=16)<br><br>Nurses (n=9)<br><br>Administrative staff (n=19)	*Shared experiences*<br>• Providers expressed the belief that integration is vital for improving patient outcomes, integration enhances the level of support they receive from colleagues, informs their clinical decision-making through consultation with providers offering different areas of expertise, builds their professional knowledge/skills over time due to these interactions, and helps prevent them from feeling overwhelmed and burned out.<br>*Shared experience challenges*<br>• A lack of shared understanding or appreciation of integration among providers created obstacles in some settings. Physicians varied considerably in the degree to which they embraced integration, some maximised opportunities to engage with behavioural health providers, whereas other physicians took a minimalist approach.<br>*Leadership*<br>• Practice leaders need to invest in staffing to coordinate the practice's integration efforts and ensure that behavioural health providers' schedules allow time for medical colleagues and new patients to access their services.<br>*Information sharing and communication*<br>• Communication strategies were used to support the delivery of integrated care (e.g. morning huddles, warm hand-offs, inviting colleagues to knock on their door).<br>• Practices used telecommunications e.g. messaging through the electronic health records system, sending group texts, telephoning, and videoconferencing to coordinate care.<br>• *Facilitator:* Physical workspaces that allow medical and behavioural health providers to work in close proximity facilitated collaboration, particularly for warm hand-offs and spur-of-the-moment consultations.

No.	Study (year)/ Setting/ Country	Research Aim(s)	Condition(s)	Psychology Professional Group	Service/intervention(s) delivered	Research Methods	Participants	Key findings (relevant to views/experiences of patients and/or professionals on psychology provision in primary care)
								• *Recommendation:* it is important that user-friendly systems for collaborating on patient care and determining when behavioural health providers are available.<br>*Practical steps*<br>• A few sites arranged blocks of time in which a behavioural health provider is on-call (rather than in scheduled sessions) to maximise their availability and enhance the timeliness and fluidity of integrated care.<br>*Challenges*<br>• High staff turnover. Challenges also resulted from limited behavioural health staffing levels that impeded patients' timely access to services.<br>• Other barriers to providing integrated care involved cultural and language differences.<br>*Recommendations*<br>• Interviewees emphasised the importance of enabling patients to meet with a behavioural health provider when they are ready (to not lose the momentum of the patient).<br>• Providers often found it beneficial to explain to patients that working with a behavioural health provider does not necessarily entail long-term, in-depth psychotherapy.<br>• Given stigmas about behavioural health services that dissuade some patients from accessing care, interviewees stressed the need for the person doing the hand-off to clearly explain the behavioural health provider's role to patients.
41.	Johanson (2021)<br><br>Primary care clinics within the FQHC (federally qualified health centre) (n=27)<br><br>USA	To survey members of a medical team on their perceptions of, attitudes toward, and use of integrated behavioural health services in a large multisite FQHC.	Behavioural health	Behavioural Health Provider (BHP)	• BHPs embedded within clinics and utilise same EHR, communication system, and support staff.<br>• BHPs work closely with primary care providers, addressing same-day mental/behavioural health concerns, conducting co-visits, flow visits (e.g., introductions to behavioural health services), and warm hand offs.<br>• Delivery of brief interventions (approximately 6x30-minute sessions).	Quantitative (survey)	Physicians, physician assistants, nurse practitioners, family nurse practitioners, nurses, medical assistants and case managers (N = 122)	• 59% strongly agreed that using BHPs improves efficiency as a healthcare provider.<br>• 83% strongly agreed that BHPs improved overall care.<br>• 72% strongly-agreed that BHPs effectively help patients address their mental health problems.<br>• 74% overall agree that BHPs effectively help patients address their physical health problems.<br>• 73% overall agree that working with BHPs increases comfort in discussing mental health issues with patients.<br>• 80% overall agree that BHPs can work effectively with other team members to address patients' physical health problems.<br>• 77% strongly agreed that BHPs are an important part of my practice.
42.	Kaitz and Ray (2021)<br><br>Traditional/coordinated, co-located, and integrated primary care practices (n = not reported)	To explore differences in providers' perspectives by provider type and level of integration.	Not reported	Psychologist	• Not reported	Mixed-methods (qualitative and quantitative survey)	Psychologists (n=30)<br><br>Primary care physicians (n=30)	• Physicians in integrated settings were the most likely to also choose integrated settings as their ideal setting,<br>• No psychologists chose coordinated/traditional settings as their ideal work setting.<br>• Psychologists currently working in integrated settings chose integrated as their ideal work setting.<br>• Physicians in traditional settings were the most dissatisfied of any other group (73%), while psychologists in integrated settings were the most satisfied (88%).<br>• The majority of psychologists in co-located and integrated settings were dissatisfied with their level of administrative support (60%).

No.	Study (year)/ Setting/ Country	Research Aim(s)	Condition(s)	Psychology Professional Group	Service/intervention(s) delivered	Research Methods	Participants	Key findings (relevant to views/experiences of patients and/or professionals on psychology provision in primary care)
	USA							• *"It is easy to communicate with my PCPs and they are open to my feedback/suggestions about patient care. The culture in general at the health centre highly values the role of behavioural health in caring for patients."* (Psychologist, Integrated)
43.	Kierans and Byrne (2010)<br><br>Not reported<br><br>Ireland	To inform the current debate relating to primary care service delivery models for mental health presentations and to contribute towards future planning.	Primary care mental health	Not reported	Not applicable	Narrative Review	Stakeholders	• Perception that patients and their carers service want a range of mental health service options (including psychological therapies) to be provided locally so they can have (improve) access to comprehensive care.<br>• View that care plans need to integrate biological, psychological, and social elements.
44.	King et al. (2013)<br><br>Divisions of General Practice (n=19)<br><br>Australia	To examine the preliminary effectiveness of an intervention delivered by specialised mental healthcare providers in an Australian primary care setting.	Suicide prevention	Fully registered mental health professional (predominantly psychologists)	• Suicide prevention services through the Access to Allied Psychological Services (ATAPS) (comparable to IAPT in the UK).<br>• Services for those who had been discharged from e.g. hospital or emergency department to GP care following a suicide attempt, or who presented to a GP after self-harm, or expressed suicidal ideation to a GP.<br>• Therapeutic support was more intensive than that provided under general ATAPS, and occurred within a 2-month time frame through an unlimited number of sessions.<br>• CBT-based treatment provided.	Mixed-methods (quantitative service data, qualitative interviews)	Mental health professionals (n=3)<br><br>Referrers (n=9, 6 GPs, 3 emergency department staff)<br><br>Divisional project officers (n=19)	• Referrers to and providers of the pilot services were generally positive, perceiving benefits to staff and patients.<br>• General practitioners and emergency department staff were happy with the referral process.<br>• Providers indicated that the pilot filled a previously unstopped gap. They highlighted that consumers were receiving an appropriate service in a more timely manner than was previously available.
45.	Klege et al. (2025)<br><br>Federally Qualified Health Centers (FQHCs) and Community Health Centers (CHCs) (n=10)<br><br>USA	To examine facilitators and barriers/challenges impacting the implementation of a Primary Care Behavioural Health (PCBH) model.	Not reported	Behavioural health consultant	• Integrating the Primary Care Behavioural Health (PCBH) model.	Qualitative (interviews)	Primary care clinician (n=7)<br><br>Behavioural health provider (n=11)	*Implementation facilitators*<br>• System and external support to implement PCBH, including resources to hire BHCs and update the EHR system.<br>• Champions in leadership positions to advocate for and monitor workflow redesign, including space allocation for BHCs.<br>• Positive provider attitudes toward organisational change.<br>• Conceptual training for all practice members, not just providers.<br>• BHCs being visible in the practice.<br>*Implementation challenges*<br>• Lack of resources, formal materials, and guidelines to continuously train new staff on PCBH.<br>• Difficulty recruiting and retaining behavioural health providers.

No.	Study (year)/ Setting/ Country	Research Aim(s)	Condition(s)	Psychology Professional Group	Service/intervention(s) delivered	Research Methods	Participants	Key findings (relevant to views/experiences of patients and/or professionals on psychology provision in primary care)
46.	Knowles et al. (2015)<br><br>Primary care practices (n= 17)<br><br>UK	To examine how the collaborative care model was implemented by usual care providers in a UK setting; and to examine how patients and providers understood and experienced the integration of mental and physical health care.	Depression in people with multimorbidity	Psychological Wellbeing Practitioner (PWP)	• Collaborative care model.<br>• 8 sessions over 12 weeks including a biopsychosocial assessment, exploration of the links between their conditions, and active treatment using a goal-oriented psychological intervention to address mental health symptoms.<br>• Treatment was chosen based on patient preference and included e.g. guided self-help, behavioural activation, graded exposure, cognitive restructuring and/or lifestyle advice.<br>• PWPs received 1 week of training from a multidisciplinary team and received 1 hour of supervision per week from their IAPT senior manager.	Qualitative (interviews)	Psychological Wellbeing Practitioners (PWP) (n=11)<br><br>Practice nurses (PN) (n=12)<br><br>GPs (n=7)<br><br>Patients (n=31)	• Patients were positive about the enhanced communication between the professionals, who typically had worked in isolation from each other.<br>• All health professionals emphasised how the new care model had enabled better liaison between themselves and supported signposting patients to a wider range of services.<br>• The collaborative care framework was valued by professionals (especially PWPs) because it increased opportunities for care co-ordination and information sharing with PNs, and also enhanced their confidence to manage mood problems in the context of complex physical symptom.<br>• The increased access to and availability of mental health care offered was considered much needed by both PNs and patients.<br>• Both PNs and PWPs also suggested that the collaborative care framework facilitated delivery of mental health care in a more acceptable, less stigmatised way.<br>• Integrated working was emphasised to be between PWPs and PNs, with GPs having relatively little involvement. Patients felt GPs would be unable to contribute due to time restrictions and preferred the closer involvement of the PN and the low intensity PWP.<br>• While patients recognised the value of seeing PWPs in the same geographic space as their nurse, (as co-location was seen to enhance care coordination and removed the stigma of accessing mental health treatment), they often stated a preference for discussing emotional health problems in a separate therapeutic space away from the nurse. Patients wanted the mental health treatment to be separate and distinct from their physical health management, and struggled with sessions that focused on dealing with their mental health condition only in the context of their physical illness.
47.	Knowles et al. (2013)<br><br>Primary care practices (n=9)<br><br>UK	To explore the extent to which collaborative care was implemented in a naturalistic National Health Service (NHS) setting.	Depression in patients with diabetes, coronary heart disease and chronic obstructive pulmonary disease (COPD)	Psychological Wellbeing Practitioner (typically employed by IAPT)	• PWPs were trained as case managers to provide brief psychological interventions for depression.<br>• Case managers in collaborative care are intended to act as 'conduits' between patients and primary and specialist care providers.	Qualitative (interviews)	PWPs (n=6) and practice nurses (n=12) at 3 months<br><br>PWPs (n= 5) and practice nurses (n=7) at 9 months.	• Co-location allows for informal collaborations and destigmatises access to mental healthcare. It provides increased co-ordination and continuity of care, and allows for greater ease of disposal for identified patients (e.g. nurses more comfortable to discuss depression with patients, as they have options available).<br>• However, there was a lack of integration (e.g. lack of shared resources, time for appointments was not allocated by practice managers, and there was limited access to practice information systems). GPs were often unaware of or uninvolved with the PWPs.<br>• Additional barriers included, role boundaries (e.g. clear division of mental and physical health work and expertise), joint meeting perceived as unnecessary, and a lack of confidence to engage in the other area of work (e.g. practice nurses not having time to discuss emotional wellbeing with patients).
48.	Koehler et al. (2020)<br><br>A family practice clinic (n=1)<br>FQHC (n=1)<br><br>USA	To investigate the patient experience of integrated behavioural health care in primary care settings.	Behavioural health issues (e.g. mental health, substance use, health behaviours, life stressors, stress related to	Integrated behavioural health care provider	• Warm-handoffs from medical providers to integrated behavioural health care providers to address behavioural health issues during primary care medical appointments and short-term behavioural health follow-up sessions.	Quantitative (questionnaires)	Patients (n=727) (family practice clinic n=546, FQHC n=181)	• Statistically significant and small to moderate direct correlation between patients' self-reported health (both physical and mental/emotional health) and their ratings of the practice as a whole (p=0.0003)<br>• Patients who rated their physical and/or mental/emotional health as better were more likely to rate their overall satisfaction with the practice higher.<br>• The results of this study suggest that primary care patients with only mild to moderate health conditions (physical and/or mental/emotional) may experience greater satisfaction with integrated behavioural health care than patients with multiple and/or severe health conditions.

No.	Study (year)/ Setting/ Country	Research Aim(s)	Condition(s)	Psychology Professional Group	Service/intervention(s) delivered	Research Methods	Participants	Key findings (relevant to views/experiences of patients and/or professionals on psychology provision in primary care)
			physical illness, poor health care utilisation)					
49.	Lorentzatou et al. (2021)<br><br>GP surgeries (n=not reported)<br><br>UK	To evaluate the implementation of a Turkish language psychotherapy programme.	Patients with complex physical and emotional needs and frequent attenders	Clinicians/ therapists from different disciplines including psychology and psychotherapy	• Community psychology, group analytic therapy, horticultural therapy, attachment theory, and dynamic therapies.<br>• Brief interventions rooted in psychodynamic/ psychoanalytic thinking.<br>• Individual and group therapy.	Narrative study (description of the psychotherapy program)	Patients and professionals (n=not reported)	• The feedback from patients and professionals showed that the program benefited the Turkish-speaking population.<br>• Patients reported feeling more able to manage their physical symptoms and mood, and that the groups helped them to develop social skills and feel confident to leave the house and use public transport.<br>• GPs reported noticing positive changes in patients' presentation and lower attendance rates.
50.	Malâtre-Lansac et al. (2020)<br><br>Physician practices (n=30)<br><br>USA	To describe factors influencing physician practices' implementation of behavioural health integration.	Not reported	Psychologist	• Co-located or collaborative behavioural health integration, with some telehealth.	Qualitative (interviews)	Practice staff and clinicians (e.g. social worker, physician, psychologist, psychiatrist, administrative) (n=47)	• Behavioural health clinicians (accustomed to 50-minute appointments and long-term patient relationships without substantial staff supervision responsibilities) could have challenges acculturating to medical clinics. This was especially true for collaborative care models that featured relatively brief patient interactions, focused on mild-moderate behavioural needs (with severe needs referred to dedicated behavioural health clinics), and that ask behavioural clinicians to supervise and assist sizeable clinical teams.<br>• Interprofessional hierarchies were another challenge. A psychologist from a large multispecialty health system explained that power differentials could reduce the effectiveness of behavioural health integration.<br>• Facilitators to resolve potential cultural mismatches included: enlisting support from organisational champions and practice leaders to increase engagement by both behavioural and non-behavioural health clinicians; interprofessional training (training all staff together) to enhance collaboration and reduce biases and stereotypes.<br>• Challenges: Practices reported that behavioural health records were shared infrequently with non-behavioural health clinicians or were accessible only with special permission. Practices reported that EHRs were "not designed for behavioural health integration" and described difficulties in sharing essential clinical information and platform interoperability (when behavioural clinicians used a different recordkeeping system).
51.	Malins et al. (2016)<br><br>GP practices (n=5)<br><br>UK	To explore the feasibility and acceptability of CBT for long-term frequent attendance in primary care and obtain preliminary evidence regarding clinical and cost effectiveness.	Frequent attenders (adults with ≥30 face-to-face GP or nurse consultations over 2 years) who often have multiple long-term conditions and psychiatric conditions	Therapist	• Participants were identified by screening patient electronic records.<br>• CBT therapy sessions (n=6-40)<br>• Patients experiencing catastrophic physical illness (e.g. cancer) or serious mental health problems (e.g. psychosis) were excluded from the study.	Mixed-methods (quantitative attendance data, qualitative analysis of semi-structured patient questionnaires)	Patients (n=32)	• Among questionnaire responders, at 12 months 88% reported satisfaction with the overall treatment offered (86% at 6 months).<br>• Participants highlighted practical strategies they had gained from therapy to cope with their difficulties.<br>• Comments from participants described the value they placed on having an opportunity to be heard and understood, without judgement. In some cases it appeared that this was the participant's main aim in therapy.<br>• If given the choice again, 88% stated that they would want the same treatment at the point it was offered. Participants focused on wishes that CBT would be made more widely and easily available, and there was a desire for greater accessibility of CBT through GP practices. Participants also described wishing that the intervention had been readily available earlier in the course of their problems.

No.	Study (year)/ Setting/ Country	Research Aim(s)	Condition(s)	Psychology Professional Group	Service/intervention(s) delivered	Research Methods	Participants	Key findings (relevant to views/experiences of patients and/or professionals on psychology provision in primary care)
52.	McElvaney and Timulak (2013)<br><br>Primary care centre (n=1)<br><br>Ireland	To investigate client experience of psychological therapy in an inner-city primary care centre.	Not reported	Senior counselling psychologist, Trainee counselling psychologist	• Brief counselling, consisting of 6-8 sessions, incorporating CBT and person-centred approaches.	Qualitative (interviews)	Patients (n=11)	• Patients found therapy to have useful aspects (e.g. feeling calm/relaxed, finding practical material helpful).<br>• Some participants noted having limited access to a therapist as an unhelpful aspect of care.
53.	Meguro (2018)<br><br>FQHC (n=1)<br><br>USA	To examine the impact of BHC services within a primary care practice in a rural Oregon county, focusing on provider satisfaction, patient satisfaction, and cost offsets.	Behavioural health services	Behavioural Health Consultant	• BHCs conduct daily chart reviews to identify patients who could potentially benefit from behavioural health services.<br>• Warm handoffs; interventions for psychosocial issues, psychoeducation, and self-management.	Mixed-methods (physician and patient satisfaction questionnaires, cost effectiveness evaluation)	Medical providers (n=9)<br><br>Patients (n=101)	• Physician satisfaction as measured by the survey was high.<br>• Generally, medical providers found BHC services to be beneficial to their patients regardless of whether the medical provider learned new techniques from the BHC or if they believed their patients perceived these services to be helpful.<br>• Patient satisfaction as measured by the survey was high. Examples of questions on the satisfaction survey include 'I learned at least one skill to help me manage my problems or concerns', 'I plan to do at least one thing differently based on what I learned today'.
54.	Miesner (2014)<br><br>Family medicine clinic (n=1)<br><br>USA	To explore patients preferred referrals process to a mental health professional within primary care (research question 2).	Anxiety, depression, bipolar disorders	Psychologist	• Not reported.	Mixed-methods (quantitative service evaluation, quantitative questionnaire)	Patients (n=36)	• Patients were asked their preferred referral process to a mental health professional, 31% endorsed 'I would prefer the physician and the mental health specialist be in the room together to talk with me on the same day as my visit with my physician', 69% endorsed 'I would prefer the mental health specialist be given my phone number and call me'.<br>• The author noted possible reasons this: it may be related to stigma; the addition of another professional discussing their mental health needs could be more intimidating; patients who participated in the study happened not to prefer the "warm handoff" option as presented in the item.
55.	Miller et al. (2023)<br><br>Primary care clinics (n=10)<br><br>USA	To examine provider satisfaction and opinions of current integrated behavioural health (IBH) process (for clinics undergoing integration).	Various (e.g. psychological disorder, health behaviour impacting healthcare such as non-compliance with medications, obesity, smoking)	Behavioural health provider (BHP)	• Behavioural health screening.<br>• Warm handoff to behavioural health provider, or scheduled for a visit.<br>• Brief targeted sessions (n=1-7) between 30-60 minutes in duration, or onward referral.	Mixed-methods (post-IBH integration survey)	Healthcare Provider Role (n=56)<br>Medical provider (n=27)<br>Patient support/ representative (n=19)<br>Registered nurse (n=2)<br>Registered dietitian (n=2)<br>Social workers (n=8)<br>Behavioural health provider (n=1)	• Provider satisfaction increased post-integration.<br>• Respondents discussed the benefits of having a BHP on-site.<br>• Respondents highlighted the competency of their behavioural health colleagues and advocated that integration of BH services "should be available for all of our patients."<br>• Respondents underscored the benefits of strong collaboration and relationships with BH practitioners contributing to care continuity and workflow.<br>• The most common concern was limited access to BHP for their patients due to staffing shortages or scheduling limitations. Logistical barriers included frustrations with connecting with BHP, difficulty scheduling appointments, and long wait times.<br>• Issues with continuity of care, including follow-up, longer-term mental and behavioural health care, social workers' role as liaisons, and overall lack of integration, were raised by several respondents.<br>• Several practitioners noted that coordination and communication systems improved through secure electronic group chats and warm telephone handoffs.<br>• Practitioners called for needed process improvements e.g. more immediate access for patients.
56.	Miller-Matero et al. (2019)	To optimise a psychological	Chronic pain	Psychologist	Proposed intervention includes:	Mixed-methods	Three focus groups (N=27)	*Qualitative*<br>• Most patients appreciated an alternative or supplement to medications.

No.	Study (year)/ Setting/ Country	Research Aim(s)	Condition(s)	Psychology Professional Group	Service/intervention(s) delivered	Research Methods	Participants	Key findings (relevant to views/experiences of patients and/or professionals on psychology provision in primary care)
	Primary care clinic (n=1)<br><br>USA	intervention for patients with chronic pain to be delivered in primary care, utilising the perspectives of providers and patients.			• Semi-structured diagnostic interview,<br>• Session 1: Psychoeducation, relaxation,<br>• Session 2: CBT,<br>• Session 3: Mindfulness<br>• Session 4: Acceptance-based strategies,<br>• Sessions last 60 minutes.<br>• Intervention to be delivered in primary care. The therapist follows a routine structure (i.e., reviewing the assignment from the previous week/assessment of the helpfulness of strategy practiced, defining new terms, assisting the patient with applying it to his or her life) and provides handouts for the patients.	(qualitative focus groups, questionnaire survey)	(psychologists (n=9), nurses and physicians (n=9), patients with chronic pain (n=9))<br><br>Focus group with psychologists from the principal investigator's institution (n=13)	• All focus group participants were in favour of offering this intervention in a primary care clinic. Participants also believed it would be helpful for the primary care providers to introduce the intervention to patients at their appointments.<br>• Primary care providers identified a lack of time during appointments as the biggest barrier to referring patients.<br>• The primary care providers were not concerned about space being an issue or inclusion of this design in the clinic workflow.<br>• Patients and primary care providers believed that the strategies across each of the sessions would be useful.<br>• Patients agreed they may better understand information if there were additional visuals included on the handouts. Psychologists suggested revisions or additions to some of the strategies. Psychologists proposed calling the assignments in between sessions something other than homework because homework may be perceived negatively.<br>• Some patients suggested incorporating a spouse, caregiver etc. in the sessions.<br>• Psychologists liked that the intervention included evidence-based strategies from multiple theoretical orientations.<br>• Patients strongly preferred in-person sessions.<br>• Regarding the frequency of sessions, the psychologists and primary care providers suggested having appointments weekly or every other week. Patients preferred biweekly sessions to allow greater time to practice in between appointments and were concerned that less frequent visits may result in forgetting to practice.<br>*Survey*<br>• 44.4% of patients (n = 4) and 66.7% of primary care providers (n = 6) stated that they appreciated a treatment for pain other than medications.<br>• Psychologists (M = 4.78) and primary care providers (M = 4.78) reported a high likelihood of referring patients to this intervention.<br>• All providers (100%, n = 18) agreed or strongly agreed that they would refer a patient to this intervention.<br>• All patients (100%, n = 9) agreed that they would participate in this intervention.<br>• Psychologists (M = 4.22), primary care providers (M = 3.78), and patients (M = 4.78) believed that the strategies in this intervention would be useful.
57.	Miller-Matero et al. (2016)<br><br>Patient centred medical home (n=1)<br><br>USA	To explore primary care physicians' beliefs regarding the benefits of integrated care for both patients and themselves.	Not reported	Psychologist, Psychology Intern	• Patients seen by a psychologist or intern immediately after a primary care physician visit in the "warm handoff" model or are scheduled for future appointments if unable to stay.<br>• Semi-structured psychological history interview and screening measures (based on referral reason).	Quantitative (questionnaire survey)	Senior staff physicians (n=15)<br><br>Residents (n=78)	• The top reasons that physicians believed their patients followed through with a visit with an integrated psychologist included that they recommended it (79.5%) and that patients can be seen in the same primary care clinic (76.9%).<br>• The overwhelming majority of physicians were satisfied with having access to an integrated psychologist (97.4%).<br>• Physicians believed that integrated care directly improves patient care (93.8%), is a needed service (90.3%), and helps provide better care to patients (80.9%).<br>• In addition, physicians reported that having an integrated psychologist reduces their personal stress level (90.1%).

No.	Study (year)/ Setting/ Country	Research Aim(s)	Condition(s)	Psychology Professional Group	Service/intervention(s) delivered	Research Methods	Participants	Key findings (relevant to views/experiences of patients and/or professionals on psychology provision in primary care)
					• Brief interventions and treatment plan recommendation provided.<br>• Feedback is communicated to the referring provider.			
58.	Monnickendam et al. (2025)<br><br>General practice (n=1)<br><br>UK	Following employment of a full-time clinical psychologist in a general practice in the UK, the research explored GPs' experience of the clinical psychologist role within their practice and what were helpful/unhelpful aspects of working together.	Not reported	Clinical psychologist	• The role of the clinical psychologist in the practice is to provide applied psychological interventions, both directly and indirectly with staff, offering consultations, training, and reflective practice.<br>• The role also involves leadership of community and systemic interventions.	Qualitative (interviews)	General practitioners (n=7)	• *Stigma.* GPs recognised that patients were more willing to access psychological support from general practice. The environment and process were less stigmatising.<br>• *Education opportunities.* Working alongside the clinical psychologist invited different types of information and bi-directional learning.<br>• *Confidence.* GPs felt more confident to discuss mental health issues with patients.<br>• *Medication.* GPs noticed a reduction in their mental health prescribing: "…my immediate feeling is that mine will be a fraction of where it was".<br>• *Workload.* The psychologist reduces workload. GPs found that psychologists sharing the workload was helpful, particularly with emotionally challenging work.<br>• *Reassurance.* There was safety with having a psychologist. A GP described the psychologist as "a safe pair of hands."<br>• *GP wellbeing.* Knowing the psychologist was available made GPs feel more relaxed, likely indirectly affecting patient care as well as potentially improving staff retention. Workplace wellbeing improved since the psychologist was introduced via a workload reduction as well as via promoting a safe, containing place to work within.<br>• *Onward referrals/other services.* GPs shared frustration and confusion in changes to mental health services and feelings of "stuckness," with not having any other option but to refer and wait on the list. Having the psychologist, GPs noted improvements in their understanding of different services, with the psychologist acting as a "pathway navigator" knowing the systems.<br>• While GPs recognised the psychologists as "experts" in mental health, this increases the risk of power imbalances and contributes to the idea that only specialists can do mental health work. It was considered that psychologists are equal to GPs: "I think because you're seen as an equal, you're not delegating down, you're delegating sideways." Psychologists are one of the clinicians as well as part of management, suggesting that psychologists have the power to influence general practice.<br>• *Community.* GPs reflected how psychologist input could impact the local community. Some noticed how psychologists introduced systemic formulations in patient care, considering the individual in the context of their experiences and relationships with others. Others highlighted how psychologists make connections throughout the community. GPs reflected on thinking holistically about patients.<br>• GPs described how the practice, and its stakeholders, had embraced the change from the exclusively GP dominant model. GPs stated psychological support should be a core part of the GP working model.
59.	Nguyen et al. (2024)	To identify strategies to successfully implement integrated behavioral	Not reported	Behavioural health consultant	• Integrated behavioral health (IBH) service, including brief interventions (designed to last	Qualitative (interviews)	Primary care clinician (n=7)	*Acceptability to IBH implementation*<br>• Implementation benefited from champions who had experience leading organisational change.

No.	Study (year)/ Setting/ Country	Research Aim(s)	Condition(s)	Psychology Professional Group	Service/intervention(s) delivered	Research Methods	Participants	Key findings (relevant to views/experiences of patients and/or professionals on psychology provision in primary care)
	Federally Qualified Health Centers (FQHCs) and Community Health Centers (CHCs) (n=10)<br><br>USA	health (IBH) into health centers by examining the IBH demonstration project in New Jersey.			15-20 minutes) with a focus on identifying feasible, behavioural changes to help with patient adherence to a care plan.		Behavioural health provider (n=11)	• The need for IBH buy-in also extended to patients - some patients have biases toward behavioural health, which a champion could address by ensuring consistent, positive messaging about IBH from all staff that the patient may encounter.<br>*Provide training that emphasises how brief behavioural health interventions differ from traditional therapy*<br>• IBH implementation requires clear understanding of how brief interventions delivered in an IBH encounter differs from traditional therapy (IBH encounter designed to last 15-20 minutes and focus on identifying feasible, behavioural changes to help with patient adherence to a care plan, it is not intended to assess and treat deeply-rooted psychological needs).<br>• Participants described that this needs to be clear to all members of the team. The primary care provider needs to describe the IBH encounter accurately to a patient during a warm hand-off. The BHC also needs to adhere to the brief intervention model to ensure their own availability for other patients and ability to meet patients' needs. Participants shared that training programs can emphasise intensive therapy, resulting in some BHCs defaulting to a traditional therapy model. IBH implementation plans should be mindful about hiring BHCs trained on brief intervention or build in IBH training and role specification.<br>*Create physical spaces for BHCs*<br>• BHCs needed physical spaces to work near the patient examination rooms so that they are readily available for patient care. The BHC needs space to prepare for new visits, document completed visits, and conduct follow-ups (sometimes done via telehealth). For some health centers, identifying space was a challenge because of limitations of the building, resulting in delayed or missed IBH encounters. When BHCs were "out of sight" from the care team, they were underutilised.<br>*Fidelity: Develop ongoing IBH training procedures for new staff*<br>• IBH training needed to start during the interview and hiring process, be ongoing, and engage multiple practice members (i.e., primary care and behavioral health clinicians).<br>*Establish scheduling systems to connect BHCs with patients*<br>• A scheduling system (planned and ad-hoc) to connect the BHC with the patient are needed to ensure patients who could benefit are not missed. Planned encounters were often identified during pre-visit planning (e.g. morning huddles), where the primary care providers flagged patients (e.g. those with chronic conditions) for the BHC to see that day. Ad-hoc encounters were those identified during the primary care visit as benefitting from IBH consultation (e.g. patient seemed anxious, had new chronic conditions). Participants shared that providers would either physically bring in the BHC during a visit or use an electronic system (chat function) to request the BHC for immediate consultation. However, BHCs were not always available ad-hoc, especially if they were seeing other patients or did not have a desk close to the examination room, which resulted in the need to schedule a separate patient appointment either in-person or via telehealth.

No.	Study (year)/ Setting/ Country	Research Aim(s)	Condition(s)	Psychology Professional Group	Service/intervention(s) delivered	Research Methods	Participants	Key findings (relevant to views/experiences of patients and/or professionals on psychology provision in primary care)
60.	Perreault et al. (2023)<br><br>University Family Medicine Group (n=1)<br><br>Canada	To examine whether a single-session intervention (SSI) increases accessibility to psychological consultations, to measure the effects of the intervention on different self-reported measures, and to assess users' consultation experiences.	Psychosocial issues	Psychologist	• SSI is a complete intervention that takes place in a single session. There is no follow-up.<br>• Key aspect is to ensure the patient leaves with a problem-solving plan and confidence in their skills/resources, knowing they can return at any time.<br>• SSIs last 60 minutes (excluding time obtaining consent and completing pre-test questionnaires). Time is spent developing the therapeutic alliance and validating suffering, defining the problem and development of possible solutions, co-editing of a personalised document and conclusion of the appointment.	Quantitative (service use data, survey)	Patients who had received the SSI (n=69)	• The SSI allowed 91% of participants to obtain an appointment in fewer than 7 working days, and only 1% waited for more than 10 days. In total, 22% received an appointment in less than one day, or on the same day as their consultation request.<br>• The no-show/late cancellation rate in previous years (2016-2018), with traditional service model, was about 22%. Implementation of SSI in 2019 reduced this rate to 10.4% during the study period.<br>• 51% of participants felt that a single session was enough for them to help solve their problem.<br>• Overall satisfaction with the consultation experience was 92.9%.
61.	Raybould (2019)<br><br>General practices (n=2)<br><br>UK	To develop a model of the social and psychological processes involved in integrating a clinical psychology service into general practice, which is grounded in the experiences of both staff and patients who have been involved in the service.	Not reported	Clinical psychologist	• One clinical psychologist working in each of the two general practices for two days per week.<br>• Consultations with rapid assessment and advice-giving.<br>• Patient consultations (typically 30 minutes).<br>• Referral to other services, and formulation and support for individuals or families identified as posing a high demand on GP resources for whom an underlying psychosocial component may be apparent.<br>• Psychological advice and consultation with other disciplines within the practices.	Qualitative (interviews)	Patients (n=9)<br><br>Administrative staff (n=4)<br><br>Practice nurse (n=2)<br><br>GP (n=4)	*Patient views*<br>• Some patients initially questioned the benefit of the service, while others invested hope *"that it would help"* and decided to *"give it a go"* and *"see what … was on offer"*.<br>• Confidence of psychology service patients developed through their experience of the sessions and related to organisational factors as well as the skills of the psychologist. Confidence also developed through allowing self-control over when/how often they used the service (e.g. one patient stated *"if I was not coping …I could always come back which was reassuring"*).<br>• Patients described not feeling rushed and finding the psychologist *"easy to talk to"* and linked this to the psychologist's knowledge. Patients appreciated working collaboratively with the psychologist, although one stated they "didn't enjoy" the sessions.<br>• Patients learned new skills, looking at problems in new ways, and developing different coping strategies. Many patients reported receiving helpful resource recommendations from the psychologist, which allowed them to help themselves outside of sessions, as well as signposting to other services.<br>• Patients reported feeling listened to but also appreciated talking to someone who "didn't just listen" but also suggested alternative options.<br>• Patients appreciated the service being local and placing it within the GP practice was important.<br>• Patients differed in their views on getting help from a new person. Some appreciated not knowing the psychologist beforehand, but others were reluctant to retell their story.<br>• Patients had differing preferences regarding the preferred age of the psychologist (one preferred the psychologist to be younger than them, another stated that they would like

No.	Study (year)/ Setting/ Country	Research Aim(s)	Condition(s)	Psychology Professional Group	Service/intervention(s) delivered	Research Methods	Participants	Key findings (relevant to views/experiences of patients and/or professionals on psychology provision in primary care)
								the psychologist to be older). One patient had a preference for the psychologist to be female.<br>• Timing was important - some patients felt their ability to engage with the psychology service was affected by their life circumstances, such as the deteriorating health of a family member.<br>*Staff views*<br>• Staff had prior experience of a pilot scheme introducing another specialist HCP not working well, and saw the psychologist as another change. Despite this, staff had a positive response, with a degree of anxiety for the service to work well. Staff described initially feeling *"nervous"* taking *"a couple of weeks…to pick up our confidence"* to use the service, and having various questions relating to *"how it would fit in"* and *"how well it would be used"*. Staff were keen to use the service to its full potential wanting to *"actively be able to use it correctly"*.<br>• Locating the psychology service within the practice helped to build the confidence of staff as it allowed them to *"get to know"* the psychologist and discuss queries and referrals.<br>• Some questioned whether the service required a clinical psychologist specifically, others felt it was important that the professional *"has the expertise"*.<br>• Being able to offer a specialist service empowered staff. Some felt that this was going *"that step further"* for patients and appreciated having *"something to offer"*, additionally perceiving that the quality of patient care had improved.<br>• Some clinicians reported that the psychology service had a practical impact on their jobs (e.g. reducing follow-up appointments, and making medication the focus of their mental health consultations), helping staff to feel effective and capable within their role. Some staff reported feeling *"less concerned"* about patients who would otherwise be waiting for another mental health service, and saw the service as taking *"some of the stress off"*. However, other staff stated that the introduction of the service did not result in any emotional impact for them.<br>• Some staff reported *"psychological strategies rubbing off"* on them and being directed to resources to use in their own work.<br>• Ratio of psychologist time/practice size: due to size differences, one practice experienced proportionately less of the service, and this was reflected in how helpful staff found it.<br>• Clinician participants differed in whether they preferred to help patients themselves in the first instance before referring to another service.<br>*Patient and staff views*<br>• Staff and patients highlighted the importance of not placing restrictions on service access, to ensure it is not difficult to get mental health support and the importance of the whole team (e.g., including reception staff) in this process. Staff and patients valued rapid access to appointments.

No.	Study (year)/ Setting/ Country	Research Aim(s)	Condition(s)	Psychology Professional Group	Service/intervention(s) delivered	Research Methods	Participants	Key findings (relevant to views/experiences of patients and/or professionals on psychology provision in primary care)
62.	Reid et al. (2020)<br><br>Primary health care teams (n=not reported)<br><br>Canada	To examine professionals' perspectives in providing children's mental health care, particularly for ongoing-complex problems.	Mental health problems in children	Psychologist	• Psychologists provided supportive care and case coordination when cases were referred outside of the practice, and also consulted on cases within the practice.	Qualitative (interview)	Family physicians (n=5), social workers (n=5), practice nurses (n=4), psychologists (n=2)	• Primary health care was seen as the place where children with ongoing-complex mental health problems would present.<br>• All participants stated they played roles in providing mental health care for this population, including assessment and treatment.<br>• Social workers and psychologists would typically receive referrals from the physician or nurse; they provided supportive care and case coordination when cases were referred outside of the practice, and also consulted on cases within the practice.<br>• Some nurses, psychologists and social workers commented that it was rare for them to provide care for young children (i.e., preschool or infants) with mental health problems.<br>• Nurses and psychologists reported their scope of practice as being limited by their regulatory body or areas of competence. One social worker and one psychologist shared they had experienced pressure to see cases that they felt were outside their expertise.<br>• Social workers and a psychologists felt that, for children with complex mental health problems, the goal should be to provide sufficient services to prevent crises.<br>• Participants identified a need for better cross sectoral integration and care coordination. All types of providers felt communication was crucial to successful collaboration.
63.	Ross et al. (2019)<br><br>Primary care practice (n=1)<br><br>USA	To evaluate the cost effectiveness of integrating behavioural health services into a primary care practice.	Behavioural health	Behavioural health clinician/ psychologist	• Brief focused therapy (e.g. 30-minutes, n=3–5 sessions).<br>• Co-consultation visits (e.g. patient visits conducted in tandem with medical provider).<br>• Warm hand-offs and support by email and phone.<br>• Facilitated referrals to community mental health providers for patients with needs that could not be addressed utilising a brief model of care.<br>• Psychologist given an office in a central location to allow medical providers to either contact electronically or in-person to assist with a patient. All information about the patient visit (e.g. the behavioural health clinician's documentation), was captured in the EHR so a visit summary could be provided.	Mixed-methods (cost-effectiveness quantitative, questionnaire survey)	Medical providers (n=13), patients (n= 29)	• The results demonstrated that integrating behavioural health services into the practice were associated with $860.16 per member per year savings or 10.8% savings in costs for patients.<br>• All medical providers (100%) were satisfied with having a behavioural health provider integrated into the practice. All providers (100%) endorsed 'the overall fit of the behavioural health provider with the team has been positive'. All providers (100%) agreed that the clinical performance of the behavioural health provider had a positive impact on the clinic's patient population.<br>• All patients (100%) agreed or somewhat agreed that the services the behavioural health consultant provided were value in helping them.

No.	Study (year)/ Setting/ Country	Research Aim(s)	Condition(s)	Psychology Professional Group	Service/intervention(s) delivered	Research Methods	Participants	Key findings (relevant to views/experiences of patients and/or professionals on psychology provision in primary care)
64.	Sallay et al. (2023)<br><br>GP clusters (n=4)<br><br>Hungary	To explore the experiences and opinions about the health psychology services (study 3).	Various (e.g. weight, smoking, stress, somatic diseases)	Health psychologist	According to the Operations Manual of the Practice Teams, the roles of the health psychologist was:<br>1. to provide individual and group therapy sessions;<br>2. to provide tailormade lifestyle counselling in areas such as stress management, weight-loss programs, and quitting smoking;<br>3. to participate in the rehabilitation of patients with somatic diseases;<br>4. to participate in prevention and screening programs.	Mixed-methods (quantitative outcome measures, qualitative focus groups)	Patients (n=21, across four focus groups)	• The health psychology services provided a pathway to new social relationships. Elderly clients and clients living with chronic diseases experienced a significant change in their relationships. Even clients living alone or with a relative suffering from chronic illness experienced a sense of community and belongingness due to the regular group activities led by the health psychologists.<br>• The experience of the health psychology consultations compensated participants for a feeling of vulnerability due to deprivation, illness, and low mood. Patients talked about newly acquired skills that helped them gain more control over their lives.<br>• There was reduced stigma in receiving psychological help.<br>• The sense of grief and loss on quality of life and health was reduced with the help of psychological interventions.<br>• Patients reported better cooperation (adherence) with their GPs.<br>• Patients regarded care as more complete and secure as a result of integration and cooperation between health professionals.
65.	Seierstad et al. (2017)<br><br>General practice (n=1)<br><br>Norway	To explore the experiences of joint consultations between GPs and CAMHS psychologists and psychiatrists.	Child and adolescent mental health care (CAMHS)	Psychologist	• Joint care discussions.<br>• Psychologist takes lead in establishing contact.<br>• The primary function of the joint consultation is to decide whether the child requires a referral or if the problem can be handled in primary care.	Qualitative (focus groups)	GPs (n=5), psychologist (n=1), psychiatrist (n=1)	• The GPs and CAMHS specialists reported that children and parents find their GP's office a safe place, whereas going to the hospital CAMHS can seem frightening to some of them.<br>• Professionals reported that families are happy that things can be solved at the practice, so they need not be referred and undergo what is often a time-consuming process.<br>• The GPs said that their diagnostic skills had improved and that they had learned how to explore for different problems, so they could better sort which patients to refer and which not to refer. The specialists agreed with the GPs self-evaluations. The GPs said they had also received concrete advice and learned strategies to use when treating such conditions as phobias and sleeping disorders.<br>• The GPs stressed the importance of meeting the CAMHS specialists in person, for learning effects, for the good of the specific patient, and for an opportunity for a short, informal consultation about other patients.<br>• Both GPs and specialists mentioned the possibility that the joint consultation could lead GPs to cut down on referrals of children and adolescents who should have been referred. Some GPs expressed discomfort with the responsibility they were expected to assume after they had decided not to refer.<br>• GPs mentioned several practical issues that were necessary to make this work in a busy practice. There must be fixed, sufficiently frequent appointments that are easy for the GPs to book. Both groups also stated that a successful collaboration depends a great deal on the specialists; not everyone would be comfortable or do a good job in such a setting.
66.	Staab et al. (2022)<br><br>Community health centres (CHC)	To provide an updated, comprehensive analysis of behavioural health integration at CHCs across the Midwest (USA) and to identify factors that distinguished CHCs	Health behaviours, mental health, substance misuse	Behavioural health provider (e.g. counsellor, psychologist)	• Brief interventions, educational materials, health behaviour counselling, short term therapy.<br>• Behavioural health and primary care services were located in the same offices at most CHCs.	Mixed-methods (quantitative and qualitative survey)	Behavioural health leaders (n=77)	• 34% indicated that limited resources were a barrier to integration. They reported that the number of behavioural health clinicians was insufficient to meet demand, and they lacked funding and space to hire additional clinicians.<br>• Respondents desired better communication between primary care and behavioural health clinicians, and better coordination with community behavioural health clinicians.<br>• They expressed a need for more integrated health information technology, particularly behavioural health documentation templates.

No.	Study (year)/ Setting/ Country	Research Aim(s)	Condition(s)	Psychology Professional Group	Service/intervention(s) delivered	Research Methods	Participants	Key findings (relevant to views/experiences of patients and/or professionals on psychology provision in primary care)
	(n=77)<br><br>USA	in the maintenance stage of integration.						• Another barrier was inadequate understanding by primary care and behavioural health clinicians of how to optimally practice in an integrated clinic. Respondents commented that primary care clinicians were sometimes resistant to changing their practices or unaware that behavioural health services were effective for improving health behaviours.<br>• Some behavioural health clinicians lacked training in integrated care or found it difficult to adhere to brief interventions while serving a high-need patient population.<br>• Patient barriers to integration were identified as: a lack of awareness, stigma, language and cultural barriers, transportation issues, and cost.
67.	Tierney and Merrick (2021)<br><br>Southcentral Foundation (SCF) health care system responsible for primary care<br><br>USA	To report on the effects of integrating behavioural health services into Southcentral Foundation (SCF) primary care clinics for Alaska Native and American Indian people in Southern Alaska.	Behavioural health	Behavioural health consultant	• BHCs are available to patients for same-day appointments, and also work with SCF's Integrated Care Teams (ICTs) as needed on patient cases.<br>• Integrated behavioural services are available to all patients, and BHCs can also be brought in at the request of a member of a patient's ICT.<br>• BHCs are often able to meet needs with brief intervention. If not, onward referrals are made.	Not reported	Patients (n=not reported)<br><br>SCF primary care employees (n=not reported)	• Integrating behavioural services into primary care improved access to services, resulting in a reduction in appointments that were not kept and greatly reduced wait times for behavioural services.<br>• 97% of patients are satisfied with the care provided by SCF.<br>• SCF has also achieved 95% employee satisfaction.<br>• SCF has sustained the integration of behavioural services for over ten years.
68.	Torrence et al. (2014)<br><br>FQHC (n=1)<br><br>USA	To assess medical personnel's attitudes and perceptions of behavioural health clinicians (BHCs) in primary care.	Medical and/or comorbid psychological conditions	Behavioural health consultant, clinical psychology practicum trainee	• Brief, short-term interventions (15-30 minutes).<br>• BHCs are embedded in the FQHC with co-located office space, integrated EHR, shared primary care receptionists, and personal communication with PCPs.	Quantitative (questionnaire)	Medical providers (n=45)	*All responses agreed that:*<br>• Using BHCs improves my efficiency as a healthcare provider (91%).<br>• Using BHCs improves my overall patient care (93%).<br>• BHCs help patients address mental health (93%) and physical health concerns (76%).<br>• Working with BHCs has increased my comfort in discussing mental health issues with my patients (73%).<br>• BHCs are an important part of my practice (91%).
69.	Turgesen (2010)<br><br>Primary care clinics (n=2)<br><br>USA	To evaluate the Integrative Primary Care Behavioral Health Program at two private primary care clinics.	Various (e.g. depression, anxiety, substance abuse)	Graduate student intern in clinical psychology	• Patients were identified by their physicians as individuals who were likely to benefit from behavioural health interventions and were referred for services.<br>• Patients received 30-minute sessions utilising evidence-based treatment practices.	Mixed-methods (routine outcome measures, questionnaire survey)	Patients (n=20)<br><br>Physicians (n=19)	*Patient satisfaction*<br>• Total scores on the Patient Satisfaction Scale ranged from 38 to 55 with a mean score of 50.1 (SD = 5.0), a maximum score of 55 was possible, indicating patient satisfaction.<br>*Physician satisfaction*<br>• 100% of physicians were satisfied with their overall experience integrating behavioural health services.<br>• Physicians agreed (100%) the services the patients received through the program were beneficial, supported their treatment plans and they received timely BHC follow-up.<br>• 100% of physicians agreed that their patients believe the services are beneficial.<br>• 58% of physicians agreed they had learned new treatment techniques through their work with the BHCs.<br>• 100% of physicians agreed that they would recommend having behavioural health consultation services to their colleagues.

No.	Study (year)/ Setting/ Country	Research Aim(s)	Condition(s)	Psychology Professional Group	Service/intervention(s) delivered	Research Methods	Participants	Key findings (relevant to views/experiences of patients and/or professionals on psychology provision in primary care)
70.	Vickers et al. (2013)<br><br>Primary care clinic (n=1)<br><br>USA	To assess the opinions and experiences of primary care providers and their support staff before and after implementation of expanded onsite mental health services and related system changes in a primary care clinic.	Various (depression, anxiety, panic disorder, social phobia, post-traumatic stress disorder)	Psychologist	• Education, support and coordination of patients' care with primary care and mental health providers.<br>• Medication and/or brief psychotherapy for panic disorder, social phobia, post-traumatic stress disorder and generalised anxiety disorder.<br>• Brief CBT in the primary care setting.	Qualitative (interviews)	Primary care providers (registered nurse n=1, physician assistant n=1, nurse practitioners n=2, clinical assistants n=2, physicians n=6) (n=12)	*Opinions of mental health services after the resource and system changes included:*<br>• Improved access to a greater breadth of mental health services onsite, and patients were able to get onsite care more quickly (reduced wait times for appointments).<br>• Providers were grateful to have mental health providers/resources within arm's length.<br>• Providers feel supported and state that team care is not just good for them, but it is the right approach for treating the whole patient.<br>• They described the process of moving patients from primary care appointments to mental health services as "smooth" and "seamless," and they place a high value on colocation and the "warm handoff."<br>• Providers said that they were making fewer referrals to outside providers after the changes were implemented. Referrals were still used, but they were more often for reasons such as patient preference or need for help with a specific disorder (e.g., eating disorders, obsessive compulsive disorder) rather than general lack of access.<br>• Providers noted that the benefit to keeping people onsite includes better coordination of care and communication than when patients are seen by a community provider.
71.	Vogel et al. (2012)<br><br>Clinical sites (n=4)<br><br>USA	To highlight best practices and common barriers to implementing an integrated primary care (IPC) model for others seeking to develop an IPC approach to training and/or practice.	Patients for whom a clinical health psychologist would be beneficial (e.g. chronic pain, panic disorder for a patient with pulmonary disorder)	Clinical health psychologist	• Hallway handoffs, same day responses to referrals, completion of initial assessments in 30 minutes, immediate verbal feedback to the referring physician, and a triage model in which appropriate patients would be offered 4–6 follow-up visits with psychology.	Case study	Psychologists (paper authors)	• Patients were triaged more effectively (no-show rates declined from 28% in 2008 to less than 10% in 2011, two years after shift to IPC). Depression (PHQ-9) and anxiety (GAD-7) scores were at the moderately severe range at baseline vs mild range at follow-up.<br>• Patient satisfaction ratings demonstrated overall positive rating of 4.08 ± 1.11 on a Likert scale ranging from 1-5 (higher values being more positive).<br>• The more physicians understood the training and expertise of clinical health psychologists, the more integrated the setting became. The more integrated the setting, the greater the respect and gratitude for how BHCs could assist physicians in meeting the primary care mission while benefiting patients considerably.<br>• Physician satisfaction was positive. Physicians perceived improved clinical effectiveness and a greater sense of competence in managing mental health conditions following implementation of IPC.<br>• Quality improvement surveys indicated that physicians appreciated the quick response of BHCs and the collaborative nature of services provided.<br>• Clinical health psychology fellows appreciated the opportunity to hone their skills in a fast-paced environment, although this was not without anxiety at times.<br>• Strategies noted to assist psychology fellows in the transition from traditional mental health practices to IPC included modelling and direct support from supervising psychologists, as well as facilitation of referrals from primary care providers to more junior members of the team.<br>• Psychology fellows identified value in working collaboratively with physicians and patients at the point of care.<br>• All psychologists recalled the need to develop more resources and skills that better fit the brief model of IPC service delivery.<br>• Almost all BHCs agreed that training in IPC was valuable, regardless of whether they intended to eventually practice in an integrated setting. Most valued was the ability to streamline the assessment/triage process, to gain knowledge about a wide variety of

No.	Study (year)/ Setting/ Country	Research Aim(s)	Condition(s)	Psychology Professional Group	Service/intervention(s) delivered	Research Methods	Participants	Key findings (relevant to views/experiences of patients and/or professionals on psychology provision in primary care)
								evidence-based interventions delivered in a short-term paradigm, and to develop skill in collaborating with physicians.<br>• High levels of mutual professional trust facilitated the transition to integrated practice.
72.	Yasmin-Qureshi and Ledwith (2020)<br><br>IAPT services across NHS Trusts (n=4)<br><br>UK	To explore South Asian women's experiences of accessing psychological therapy.	Mild-moderate depression and anxiety	Therapist or counsellor	• CBT or counselling within a service that offers an IAPT model.<br>• Delivered at GP practices or community buildings.	Qualitative (interviews)	Patients (n=10)	• All participants reported that the IAPT service was easily accessible due to the location.<br>• Some participants reported reluctance from the GP to make a referral for psychological therapy even when they asked to be referred.<br>• 8/10 participants reported a long waiting period between having their assessment and having their first one-to-one therapy appointment. Waiting for therapy often led to the development of unhealthy coping mechanisms<br>• There was also little control over what treatment interventions clients received, as interventions were based on the severity of mental health difficulties, which did not always meet the expectations of the participant.<br>• Although manualised CBT treated mental health symptoms, CBT and the IAPT model was perceived by some to be a Eurocentric model that does not cater to issues faced by South Asian women, even if manualised CBT was delivered by a South Asian therapist.<br>• Most therapists were described as knowledgeable, warm, trusting, empathic, non-judgmental and as good listeners and good at offering participants practical advice to manage their mental health. Three participants found their therapists inflexible and rigid; this appeared to be mediated by the rigidity of the manualised approach.

Abbreviations: BHC = behavioural health consultant; BHP = behavioural health provider; CAMHS = child and adolescent mental health services; CBT = cognitive behavioural therapy; CHC = community health centres; COPD = chronic obstructive pulmonary disease; DBT = dialectical behaviour therapy; EHR = electronic health record; fACT = focused acceptance and commitment therapy; FQHC = federally qualified health centre; GP = general practitioner; GPCP = general practitioner clinical psychologist; IAPT = Improving Access To Psychological Therapies service; IBH = integrated behavioural health; ICT = integrated care teams; IPC = integrated primary care; MCI = multicomponent intervention; NHS = National Health Service; PCBH = primary care behavioural health; PCNs = primary care networks; PCPCS = primary care psychotherapy consultation service; PN = practice nurse; SBIRT = screening, brief interventions, and referral to treatment; SSI = single-session intervention; T/APPS = trainee associate psychological practitioners; t-CBT = telephone-based CBT
